# Summarizing and exploring data of a decade of cytokinin-related transcriptomics

**DOI:** 10.3389/fpls.2015.00029

**Published:** 2015-02-17

**Authors:** Wolfram G. Brenner, Thomas Schmülling

**Affiliations:** Dahlem Centre of Plant Sciences, Institute of Biology/Applied Genetics, Freie Universität BerlinBerlin, Germany

**Keywords:** cytokinin, *cis* element, gene regulation, hormone action, meta-analysis, regulatory network, signal transduction, transcriptomics

## Abstract

The genome-wide transcriptional response of the model organism *Arabidopsis thaliana* to cytokinin has been investigated by different research groups as soon as large-scale transcriptomic techniques became affordable. Over the last 10 years many transcriptomic datasets related to cytokinin have been generated using different technological platforms, some of which are published only in databases, culminating in an RNA sequencing experiment. Two approaches have been made to establish a core set of cytokinin-regulated transcripts by meta-analysis of these datasets using different preferences regarding their selection. Here we add another meta-analysis derived from an independent microarray platform (CATMA), combine all the meta-analyses available with RNAseq data in order to establish an advanced core set of cytokinin-regulated transcripts, and compare the results with the regulation of orthologous rice genes by cytokinin. We discuss the functions of some of the less known cytokinin-regulated genes indicating areas deserving further research to explore cytokinin function. Finally, we investigate the promoters of the core set of cytokinin-induced genes for the abundance and distribution of known cytokinin-responsive *cis* elements and identify a set of novel candidate motifs.

## Introduction

Cytokinins are a class of plant hormones with a broad range of functions in regulating plant growth and development as well as physiologicial processes (Werner and Schmülling, 2009; Kieber and Schaller, 2014). In *Arabidopsis thaliana*, they are perceived by three closely related receptors localized mainly at the ER membrane (Heyl et al., 2012). Perception of cytokinins by the receptors triggers a multistep His-Asp phosphorelay signal transduction chain transducing the signal to the nucleus, where a set of MYB-like transcription factors, the type-B response regulators, are activated to induce the transcription of their target genes (Sakai et al., 2000; Hwang et al., 2002). Among the direct targets are type-A response regulator genes, which encode negative regulators of the upstream signal transduction system (Hwang and Sheen, 2001). Additionally, a set of transcription factors of the ERF/AP2 family is involved in mediating the response to cytokinins, named cytokinin response factors (CRFs) (Rashotte et al., 2006).

The genome-wide transcriptional response of *Arabidopsis thaliana* to cytokinins was investigated by different research groups as soon as large-scale transcriptomics became affordable. The flood of data started in 2002 with a publication addressing shoot induction in calli by cytokinin (Che et al., 2002), followed by two studies dedicated to cytokinin induction in seedlings (Rashotte et al., 2003; Brenner et al., 2005), the latter using the full genome ATH1 GeneChip®, and a study focusing on the response regulator ARR22 (Kiba et al., 2004). Since then, many more large-scale transcriptomic datasets related to cytokinin have been generated using different technological platforms, some of which are published only in databases (Brenner et al., 2012), culminating in an RNA sequencing experiment (Bhargava et al., 2013). All of the microarray studies, however, suffer from the noisy nature of the data generated by this hybridization-based method, potentially resulting in a substantial number of false positives and negatives. An attempt to identify a core set of cytokinin-regulated genes has been made using a single microarray dataset and applying elaborate statistical methods in order to narrow down the number of genes (Nemhauser et al., 2006). This attempt, however, relying on only one single microarray experiment, was tained with the inevitable insecurities inherent to the technology and prone to contain false positive genes. The obvious solution to overcome this problem is to extend the database. Meta-analysis, the statistical summary of many studies on the same subject, can be used to increase the statistical power beyond that of individual studies (De Magalhães et al., 2009; Plank et al., 2012). Two approaches to establish a core set of cytokinin-regulated transcripts by meta-analysis of transcriptomic data using different preferences regarding the datasets used have been published (Brenner et al., 2012; Bhargava et al., 2013).

The nature of the promoters of cytokinin-inducible genes has drawn interest since the discovery that type-B response regulators realize the immediate early transcriptional output of the cytokinin signal transduction chain. The sequence (A/G)GAC(C/T) was identified as the ARR1-binding DNA motif (Sakai et al., 2000, 2001), which was later named cytokinin response motif (CRM) (Ramireddy et al., 2013). The CRM was more precisely characterized leading to the octameric motif AAGAT(C/T)TT (Taniguchi et al., 2007) named extended CRM (ECRM) by Ramireddy et al. (2013). Based on this knowledge, the promoter of *ARR6* was thoroughly investigated, and it was found that all of the four ECRM variants within 350 bp upstream of the transcription start site contribute to a different degree to the promoter's cytokinin responsiveness and to its response toward different type-B response regulators (Ramireddy et al., 2013). However, it was noted that the promoters of numerous known cytokinin responsive genes lack an ECRM. It may be that functional ECRM variants are located further upstream than the −1000 bp sequences analyzed or within the transcribed region of the genes. However, since most functionally relevant *cis*-elements are located within a distance of −500 bp upstream of the transcription start site (Franco-Zorrilla et al., 2014), we would expect that this is not often the case. This observation may rather suggest that there must be additional DNA sequences mediating a cytokinin response.

In this publication, we add another meta-analysis of cytokinin response genes derived from an independent microarray platform. All the available meta-analyses and the RNA sequencing data have been compared to establish an advanced core set of cytokinin-regulated transcripts. In addition, the identification of evolutionary conserved regulation of orthologous rice genes by cytokinin is reported. The identity of these genes indicates promising directions of future research into areas which have not yet been functionally linked to cytokinin activity. The promoter sequences of core set genes have been analyzed leading to the proposal of putative novel cytokinin response motifs.

## Materials and methods

### CATMA microarray meta-analysis

CATMA microarray hybridization and data processing were described before (Brenner and Schmülling, 2012; Ramireddy et al., 2013). Meta-analysis of the set of four datasets, two of which were published (Brenner and Schmülling, 2012; Ramireddy et al., 2013) was performed as described (Brenner et al., 2012). Calculation of average expression values, sorting and filtering of the data were carried out in Microsoft Excel. The individual datasets are accessible in the ArrayExpress database (http://www.ebi.ac.uk/arrayexpress/) under the accessions E-MEXP-670, E-CAGE-111, E-MEXP-689, and E-CAGE-49.

### Comparative overlap analysis of large-scale transcriptomic datasets related to cytokinin

Four data sources were used for the comparative analysis: (1) The meta-analysis of CATMA microarray results presented in this study; (2) a meta-analysis of publicly available datasets performed with the Affymetrix ATH1 gene chip focusing on studies with a similar experimental layout (Brenner et al., 2012); (3) a meta-analysis of publicly available datasets performed with the Affymetrix ATH1 gene chip based on a more broadly selected dataset (Bhargava et al., 2013); and (4) a global gene expression analysis in response to cytokinin using RNA sequencing (Bhargava et al., 2013). The datasets were compared using an overlap analysis, and the results were plotted as Venn diagrams.

### Computational analysis of annotated gene functions

Quantification of the major GO categories was carried out using the GO annotation search, functional categorization and download service at TAIR (http://www.arabidopsis.org/tools/bulk/go/index.jsp). Detailed GO term enrichment was carried out using the AmiGO tool (http://amigo1.geneontology.org/cgi-bin/amigo/blast.cgi).

### Promoter analysis

The −1000 bp sequences of the advanced core set of cytokinin-induced genes were downloaded from TAIR using the bulk data retrieval tool (http://www.arabidopsis.org/tools/bulk/sequences/index.jsp). Motif mapping was carried out using the Toucan2 suite of sequence analysis tools (https://gbiomed.kuleuven.be/apps/lcb/toucan/toucan.jnlp) (Aerts et al., 2003, 2005). Degenerated motifs were counted with the help of a self-made program written in Microsoft Visual Basic for Applications (Supplementary Text). Statistical evaluation of these results were carried out using Microsoft Excel. The consensus logo of the motifs found was generated using the weblogo service at Berkeley university (http://weblogo.berkeley.edu/) (Schneider and Stephens, 1990; Crooks et al., 2004).

### Analysis of cytokinin-related transcriptomic data from rice

The raw data of three transcriptomic datasets from rice with the accessions GSE6719, GSE37557, and GSE55902 were downloaded from the GEO database (http://www.ncbi.nlm.nih.gov/geo/). GSE6719 contains data from root and leaf samples of the cultivar Nipponbare treated with 5 μM *trans*-zeatin for 30 or 120 min, respectively. GSE37557 is a series of different hormone treatments, from which we selected only the data from cytokinin treatment which were obtained from 7-day-old seedlings of the cultivar IR64 treated with 50 μM benzyl adenine (BA) for 3 h. The data of GSE55902 are from samples of the third leaf of 30-day-old plants of cultivar Nagina22 treated for 72 h in darkness with 2 mg/mL BA. The data were normalized with the RMA algorithm using the Bioconductor affy package (Gautier et al., 2004; Gentleman et al., 2004). Fold-changes, *p*-values and FDR-corrected *q*-values (Benjamini and Hochberg, 1995) were calculated using Microsoft Excel. Significantly differentially expressed genes (differential expression ≥ 2-fold, *q* ≤ 0.05) were also identified in Excel. One dataset (GSE37557) gave no meaningful data for the cytokinin-induced samples. Therefore, the analysis was continued with the two remaining datasets and no meta-analysis was performed with the rice data.

### Identification of rice orthologs of arabidopsis genes and determination of their transcriptional regulation by cytokinin

We employed two methods for the identification of rice orthologs of Arabidopsis genes. Firstly, we used reciprocal BLAST, where in the first step the rice database was queried with Arabidopsis protein sequences. In the second step, the Arabidopsis database was queried with the top results of the first search until the original Arabidopsis protein was no longer the top hit of the resulting list. All rice genes corresponding to those proteins yielding the Arabidopsis protein encoded by a cytokinin-regulated gene as the most closely related rice counterpart were classified as orthologs. Secondly, we used the service for retrieving rice orthologs for Arabidopsis genes (http://www.rothamsted.ac.uk/ppets/modperl/ets2/at_template_form.pl?config=Wheat) at PpETS (http://www.rothamsted.ac.uk/ppets/modperl/index.pl) (Mitchell et al., 2007). We combined the partially overlapping (20%) results of both searches and analyzed the cytokinin response of these genes in the microarray data described above.

## Results and discussion

### Meta-analysis of cytokinin-related transcriptomic data collected by the CATMA microarray

The CATMA microarray is a cDNA microarray designed by a European consortium in the early 2000s to achieve the best possible gene coverage (Crowe et al., 2003; Hilson et al., 2003). To achieve this, gene-specific probes—so-called GSTs (gene-specific tags)—were identified bioinformatically to cover the region of each transcript that is least similar to any other transcript in the transcriptome. These regions were amplified by PCR using a two-step process and spotted onto microarrays. The advantage of this microarray over the popular and widely used Affymetrix ATH1 array is that it is based on a more advanced genome annotation called EuGène (Schiex et al., 2001), therefore containing an improved representation of the Arabidopsis transcriptome. Its competitive performance in comparison to the Affymetrix and Agilent platforms has been demonstrated (Allemeersch et al., 2005). Cytokinin-related transcriptome studies using the CATMA microarray have been published before (Heyl et al., 2008; Brenner and Schmülling, 2012; Ramireddy et al., 2013), but the full amount of cytokinin-related data retrieved from this technology has not yet been evaluated. To get an overview over the full dataset we conducted a meta-analysis using the same approach as used before to evaluate publicly available ATH1 array data (Brenner et al., 2012). The top 25 induced transcripts are shown in Table 1 and the result of the entire analysis is available in Supplementary Table 1. A number of previously unknown genes was found to be regulated by cytokinin in the CATMA microarray meta-analysis, which, due to the statistical power of four summarized microarray studies represents a robust dataset. The result of this meta-analysis will be compared with results obtained the ATH1 microarray and RNA sequencing data in the following sections.

**Table 1:** **Top 25 cytokinin-induced genes from a meta-analysis of transcriptomic data obtained with the CATMA microarray**.

**CATMA ID**	**AGI**	**Gene symbol**	**Description**	**Avg. ratio BA15 vs. BA0**	**Avg. ratio BA120 vs. BA0**	**Avg. ratio BAx vs. BA0**	**Meta statistics**
CATMA5a58475	AT5G62920	*ARR6*	Type-A response regulator 6	4,83	3,08	3,96	129,82
CATMA3a37230	AT3G44326		F-box family protein	2,44	2,42	2,43	94,11
CATMA4a31370	AT4G29740	*CKX4*	Cytokinin oxidase 4	7,60	6,29	6,95	86,41
CATMA1a18090	AT1G19050	*ARR7*	Type-A response regulator 7	11,72	8,94	10,33	85,09
CATMA5a43950	AT5G47980		HXXXD-type acyl-transferase family protein	2,00	12,29	7,15	80,18
CATMA1a57750	AT1G68360		C2H2 and C2HC zinc fingers superfamily protein	3,02	2,51	2,77	77,01
CATMA5a17520	AT5G19110		Aspartyl protease family protein	2,01	4,48	3,25	76,89
CATMA2a38980	AT2G40670	*ARR16*	Type-A response regulator 16	2,39	7,51	4,95	75,36
CATMA4a31320	AT4G29690		Alkaline-phosphatase-like family protein	2,10	6,76	4,43	67,16
CATMA4a25570	AT4G23750	*CRF2*	Cytokinin response factor 2	2,01	2,58	2,30	64,72
CATMA4a38980	AT4G37410	*CYP18F4*	Member of CYP81F	2,63	7,58	5,11	56,62
CATMA4a31330	AT4G29700		Alkaline-phosphatase-like family protein	2,26	4,56	3,41	50,84
CATMA2a00940	AT2G01890	*PAP8*	Purple acid phosphatase 8	2,12	3,12	2,62	44,09
CATMA1a16200	AT1G17190		Glutathione S-transferase tau 26	2,50	3,33	2,91	43,79
CATMA2a39655	AT2G41310	*ARR8*	Type-A response regulator 8	2,68	4,50	3,59	42,97
CATMA4a25920	AT4G24190	*SHD*	HSP90-like protein SHEPERD	2,08	2,91	2,50	39,78
CATMA1a41350	AT1G50280		Phototropic-responsive NPH3 family protein	5,76	9,11	7,44	39,67
CATMA1a44090	AT1G53060		Legume lectin family protein	2,12	16,16	9,14	34,57
CATMA1a26300	AT1G28100		Unknown protein	2,83	3,80	3,31	33,72
CATMA3a52020	AT3G58990	*IPMI1*	Isopropylmalate isomerase 1	3,86	2,22	3,04	32,95
CATMA5a61570	AT5G66210	*CPK28*	Calcium-dependent protein kinase family protein 28	2,71	2,13	2,42	32,62
CATMA1a17855	AT1G18800	*NRP2*	NAP1-related protein	2,28	2,54	2,41	32,48
CATMA5a37190	AT5G41590		Protein of unknown function (DUF567)	2,13	2,18	2,15	32,40
CATMA3a18390	AT3G18773		RING/U-box superfamily protein	4,20	4,19	4,20	31,55
CATMA5a07985	AT5G08640	*FLS1*	Flavonol synthase 1	3,80	6,57	5,19	31,15

*The results of four different microarray studies (see Materials and Methods) were processed to calculate Meta statistics values using R as described in Brenner et al. (2012). In all of these experiments, seedlings were grown for 5–7 days in liquid medium (1/2 MS, 1 g/L sucrose, 0.5 g/L MES, pH 5.7) before they were induced with 1 or 5 μM of the synthetic cytokinin 6-benzyladenine (BA) for defined time periods. The labels BA0, BA15, BA120 correspond to 0, 15 or 120 min of induction, respectively. This table shows the top 25 genes of Supplementary Table 1E; the complete result can be viewed in Supplementary Table 1*.

### Comparison of recent meta-analyses and large-scale RNA sequencing datasets

**Figure 1:**
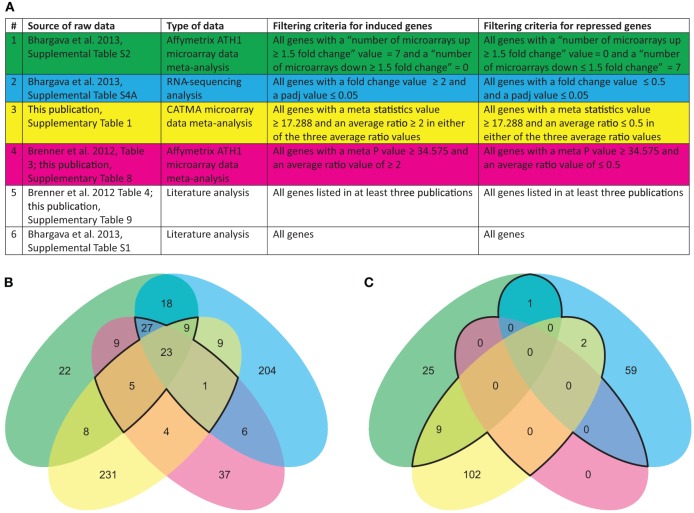
**Data sources and Venn diagram summarizing large-scale transcriptomic studies related to cytokinin. (A)** Source and type of data reporting cytokinin-regulated gene expression used in this study. Overlap analysis of cytokinin-induced **(B)** and cytokinin-repressed **(C)** genes identified in the different studies. The color code of the circles in **(B,C)** corresponds to the code in the table shown in **(A)**. The core sets of regulated genes are encircled with a black line.

Each of the technological platforms used for obtaining transcriptomic data—Affymetrix microarrays, CATMA microarrays, and RNA sequencing—has its specific benefits and weaknesses. In order to reduce biases introduced by the technologies themselves, we compared the datasets listed in Figure 1 using an overlap analysis (Figure 1).

The first obvious observation is that both the RNA sequencing data and the CATMA data contain many unique genes (68 and 80%, respectively) and have a small overlap (<11%) whereas the two datasets from meta-analyses of Affymetrix ATH1 data have a much larger overlap (64% of genes detected in either meta-analysis) and contain only 18 and 33% unique genes, respectively. The mismatch between the results of the two microarray platforms is only to a small part due to a lacking overlap in the gene sets: 61% of the genes found regulated in the analysis of the Affymetrix data also have a probe on the CATMA array, and 90% of the genes found regulated on the CATMA array have a probe set on the Affymetrix array. The mismatch may rather demonstrate a weakness of quantitative hybridization-based methods, in which the sensitivity for detecting a given transcript is partly dependent on the sequence of the probe.

The outcome of this comparison demonstrates that the choice of technology is an important determinant of the final outcome of a large-scale transcriptomics experiment. Furthermore, it shows that the preselection of primary datasets for a meta-analysis—reduction of the data to a set of comparable growth conditions, treatments, and time points (Brenner et al., 2012) vs. expansion to multiple conditions (Bhargava et al., 2013)—has only a relatively small impact on the outcome, at least in this particular case.

### Determination of a core set of cytokinin-regulated transcripts

Sixty five transcripts (12% of all transcripts listed in the source datasets) were found to be induced in at least three of the four independent analyses (the area encircled with a black line in Figure 1B), and 23 (3.6%) in all four. These transcripts can be referred to as the core set of cytokinin-induced transcripts. All transcripts that were positive in at least three of the four datasets shown in Figure 1 are listed in Table 2A which includes, in addition, also data from literature analyses. The entire list of cytokinin-induced transcripts alongside with information in which datasets they appear can be viewed in Supplementary Table 2A.

**Table 2:**
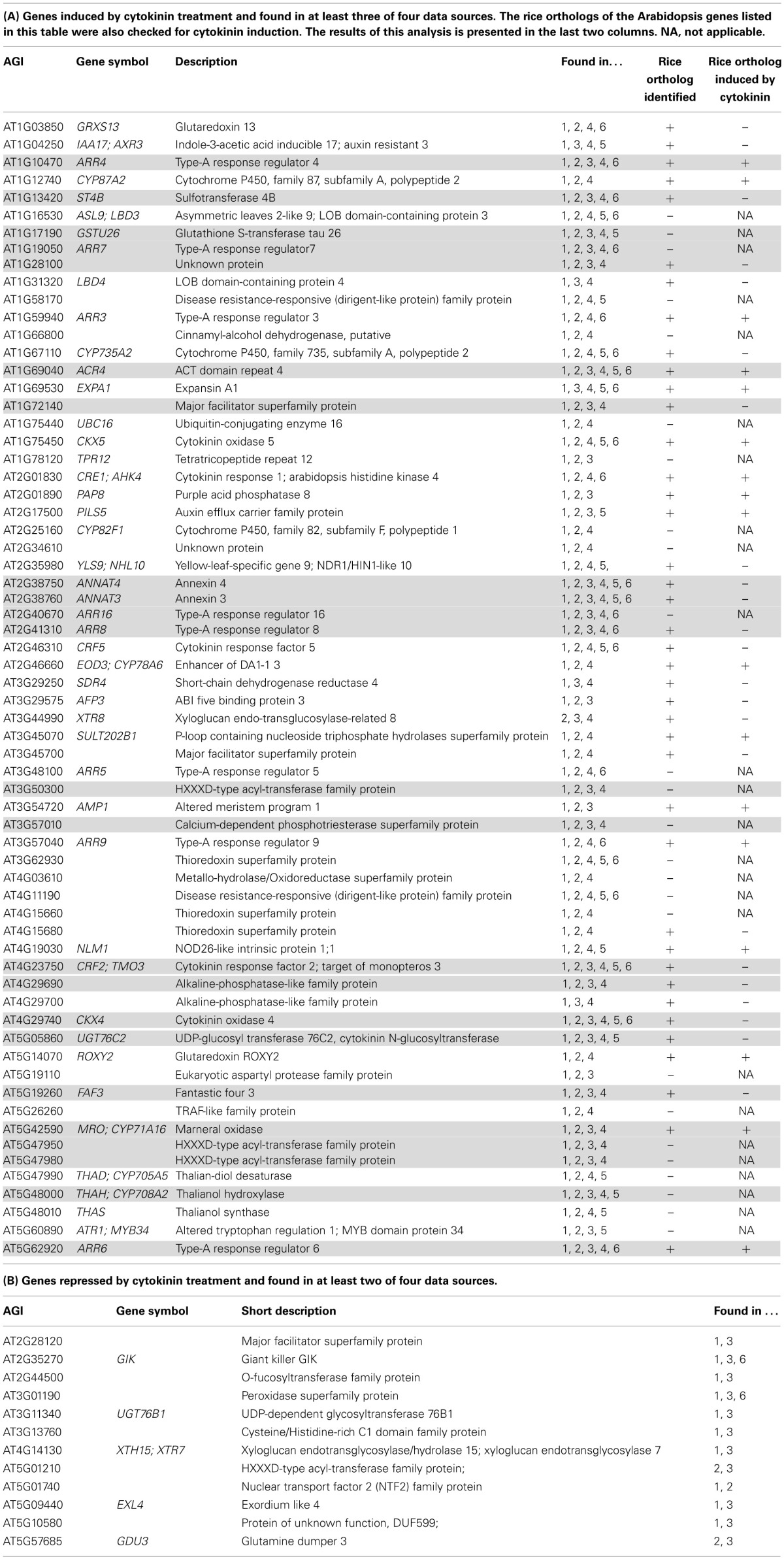
**Advanced core set of cytokinin-regulated genes**.

This list of genes is the result of the intersection analysis using Venn diagrams as detailed in Figure 1. The numbers in the last column refer to the data sources listed in Figure 1. Genes highlighted in gray were found in all four data sources shown in Figure 1. The genes are sorted according to their AGI. The results of literature analyses (no. 5 and 6) have been included in the table as additional information.

The list describing the advanced core set of cytokinin-induced genes is with 65 members shorter than most other lists of cytokinin-induced genes in meta-analyses published here (Supplementary Tables 1, 9) and in previous investigations (Brenner et al., 2012; Bhargava et al., 2013) or individual microarray studies referenced therein. This is due to the application of the most stringent selection criteria: Firstly, the high ratio of false-positive detection of individual microarray experiments was already reduced by combining several of these in the meta-analyses used as the basis for this study. Secondly, variation by technology (different microarray platforms and RNA sequencing, respectively) or different selection of original datasets for meta-analysis was eliminated by comparative overlap analysis represented as a Venn diagram in Figure 1. This way, the genes presented here—especially those found in all four source datasets—represent the most robust cytokinin response genes. A possible drawback of this stringent method is that genes, which are only present on one of the microarray platforms are wrongly eliminated as false negatives. This could particularly be the case for genes prone to high transcriptional variation due to signaling inputs other than cytokinin or for genes missing on one of the microarray platforms. However, notably a significant portion (57%) of a list of 14 cytokinin marker genes (Nemhauser et al., 2006) are present in the advanced core set.

No common cytokinin-repressed transcripts were found in all four independent analyses. This was inevitable because in one of the meta-analyses no gene was found that matched the cut-off of an average fold-change =0.5 (Brenner et al., 2012). But surprisingly, there were also no cytokinin-repressed transcripts consistently found in all three remaining analyses. Therefore, we assembled all transcripts found in only two independent analyses as cytokinin-repressed transcripts (the area encircled with a black line in Figure 1B) in Table 2B. The entire list of cytokinin-repressed transcripts alongside with information in which datasets they appear can be viewed in Supplementary Table 2B. Because these genes were less well supported than the cytokinin-induced genes we focused for the following analyses only on the latter category.

### Comparisons of cytokinin-induced arabidopsis genes with their rice orthologs

We made an attempt to find evolutionary conserved cytokinin-induced genes by analyzing the cytokinin response of rice orthologs of regulated Arabidopsis genes in the available transcriptomic data from rice. Only two suitable datasets from rice could be used for this analysis, therefore the rice data lack the breadth sufficient to conduct a meaningful meta-analysis. For this reason, we made a direct comparison with these two datasets.

For 40 of the 65 cytokinin-induced Arabidopsis genes (62.5%) at least one rice ortholog was identified (Supplementary Table 3). 17 of these genes (42.5%) have at least one rice ortholog that is also induced by cytokinin. Besides genes of the cytokinin metabolism and signaling circuitry, such as those encoding type-A response regulators, cytokinin receptors, and enzymes involved in cytokinin metabolism, several other orthologous genes with other interesting functions are also induced by cytokinin in rice. *ACR4*, *EXPA1*, and *AMP1* are discussed in more detail further below. Other genes include *CYP87A2*, encoding a cytochrome P450 with unknown function, the purple acid phosphatase gene *PAP8*, *PILS5* encoding a member of a recently characterized novel auxin efflux carrier protein family (Barbez et al., 2012; Dal Bosco et al., 2012; Feraru et al., 2012), a flavonoid sulfotransferase gene (*SULTR202B1*) (Hashiguchi et al., 2013), the aquaporin gene *NLM1*, the glutaredoxin gene *ROXY2*, a gene encoding a cytochrome P450 enzyme required in the sporophytic tissue of the mother plant to promote seed growth (*EOD3*) (Fang et al., 2012), and the gene encoding the marneral oxidase *MRO*. The evolutionary conserved induction of these genes by cytokinin in both distantly related species suggests a functional relevance of this regulation. We anticipate that analysis of the transcriptomic response to cytokinin in rice covering more tissues, developmental stages and time points would reveal additional orthologous genes regulated by cytokinin which went undiscovered in the limited data set currently available.

### Functions of the core set of cytokinin-regulated transcripts

**Figure 2:**
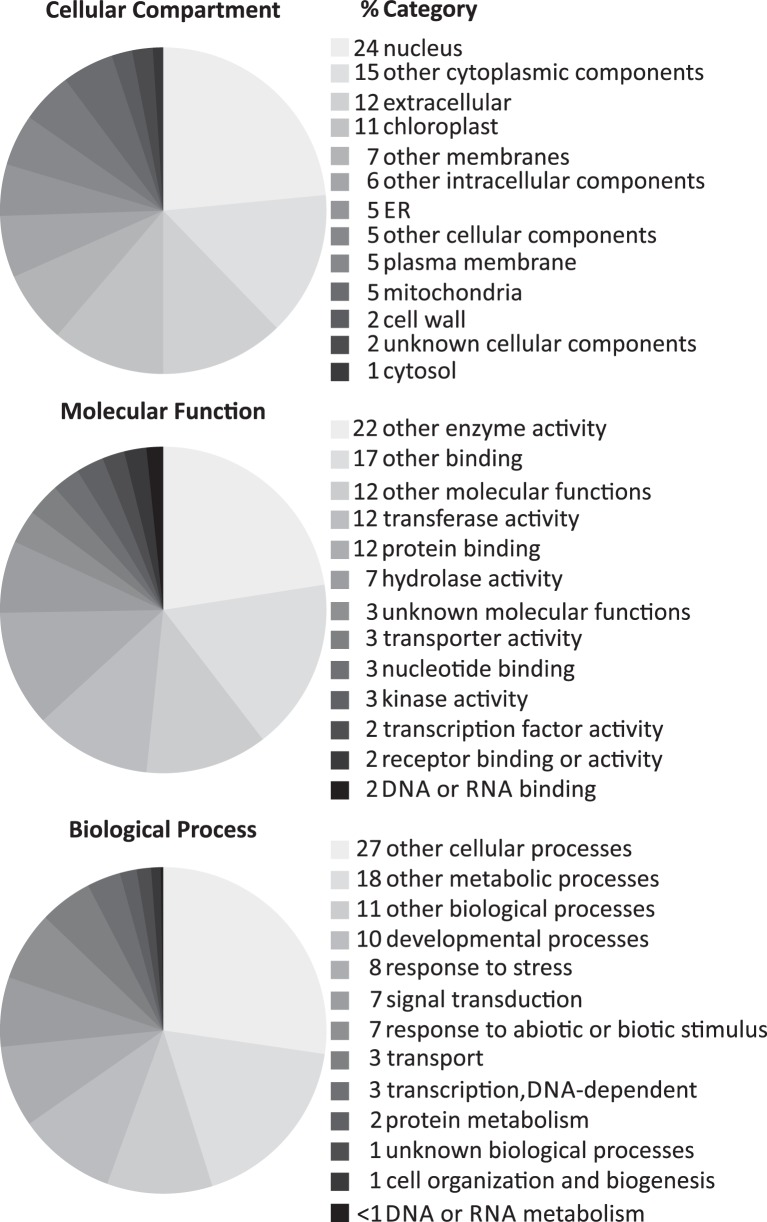
**Annotated subcellular localizations, molecular functions and biological processes of the core set of cytokinin-induced genes**. The genes listed in Table 2A were evaluated with regard to their GO categorization using the GO categorization tool provided by TAIR (http://www.arabidopsis.org/tools/bulk/go/index.jsp), which matches a given set of genes with a reduced set of basic GO terms (GOslim). The resulting table was imported into Microsoft Excel for generating the cake diagrams. The Excel diagrams were reformatted using CorelDRAW to fit them into the frame of a figure.

To get a first insight into the functions of the core set of cytokinin-regulated transcripts, we carried out a rough functional categorization of the genes, using the GO functional categorization tool at TAIR, which uses a set of less specialized GO terms (GOslim). Remarkably, the molecular functions and biological roles of more than half of the cytokinin-regulated genes do not belong to one of the major categories (Figure 2). The three major categories of molecular functions were transferase, protein binding and hydrolase activity. Relatively few genes are candidates for downstream signaling processes (transcription factors, protein-modifying or degradation processes). The majority of the genes characterized to be involved in a biological process play a role in developmental processes, responses to stress, signal transduction, and responses to abiotic and biotic stimuli, consistent with previous knowledge about cytokinin action (Werner and Schmülling, 2009; Kieber and Schaller, 2014).

To investigate the functional categories of cytokinin-induced genes in more detail, we carried out a statistical analysis of the abundance of the GO terms associated with the corresponding genes using AmiGO (Carbon et al., 2009) (Supplementary Figure 1 and Supplementary Table 4). Unsurprisingly, GO categories associated with hormone—in particular cytokinin—action and metabolism were most strongly enriched among the cytokinin-induced transcripts. Other notable overrepresented GO terms are associated with circadian rhythm, flavonoid—in particular anthocyanin—metabolism, and thalianol metabolism. Additionally, a substantial fraction of the encoded proteins have glutaredoxin/arsenate reductase activity. Due to the small number of cytokinin-repressed transcripts, the GO term enrichment analysis did not return any useful results for downregulated genes (data not shown).

For more than two thirds of the core set of cytokinin-induced genes (Table 2)—40% of the genes detected in at least three large-scale analyses and 35% of the 23 genes detected in all four data sources—no published data regarding their functional characterization exist. A notable group among these consists of three genes encoding HXXXD-type acyl transferases, two of which are closely related; the third one is encoded on the same operon-like mRNA as the cytokinin-regulated genes encoding three thalianol metabolic enzymes THAS, THAD, and THAH (Brenner et al., 2012). Also, there are two closely related (and genomically neighboring) genes encoding alkaline phosphatase-like family proteins, which have thus far not been studied. The same is true for three thioredoxin genes. These genes are prime candidates to investigate in order to unravel so far unknown connections between the immediate early cytokinin signaling chain and gene expression on the one hand and the pleiotropic physiological and phenotypical output on the other hand.

The roles of many of the already functionally characterized genes were discussed in previous articles (Brenner et al., 2005, 2012; Brenner and Schmülling, 2012; Bhargava et al., 2013; Ramireddy et al., 2013), such as auxin- and redox-related genes as well as genes involved in cytokinin signaling and homeostasis. Other genes with known function, however, were not discussed in detail. In the following sections we present and discuss a few of these as they might indicate underexplored areas of cytokinin research.

One remarkable group of genes is represented by *expansin A1* (*EXPA1*): A conspicuous number of expansin genes is regulated by cytokinin in individual experiments (data not shown), but only *expansin A1* is consistently upregulated throughout all of them. Expansins, encoded by a family of at least 36 genes in Arabidopsis (Sampedro et al., 2006), are proteins capable of loosening the cell wall in a pH-dependent manner without having to cleave covalent molecular bounds (Cosgrove, 2000; Durachko and Cosgrove, 2009), probably by enabling slippage between cellulose microfibrils and matrix glucans under acidic conditions (Wei et al., 2011). Therefore, they are thought to be the major players in short-term cell wall loosening (Cosgrove, 1999) excerting functions in cell growth or differentiation processes. Another cytokinin-induced gene coding for a cell-wall modifying enzyme, a xyloglucan endo-transglucosylase, is encoded by *XTR8*. Since cytokinin is involved in regulating growth processes and growth involves the irreversible increase of the individual cell volume requiring plastic stretching of the cell wall, it would be worth investigating whether at least part of the cytokinin-mediated growth response is mediated by modulation of expansin gene expression.

Two of the 17 known sulfotransferase genes in Arabidopsis (Klein and Papenbrock, 2004) are induced by cytokinin, *ST4B* and *SULT201B1*. The latter sulfotransferase acts on flavonoid glucosides (Hashiguchi et al., 2013), while the substrate of the former sulfotransferase is not known. Flavonoids are inhibitors of basipetal auxin transport in roots (Brown et al., 2001). As it is thought generally that sulfatation modulates the biological activity of compounds by increasing their water solubility (Hashiguchi et al., 2013) it is tempting to speculate that cytokinin could act indirectly on polar auxin transport by modification of the biological activity of flavonoids through modulation of sulfotransferase activity.

Annexins are a family of Ca^2+^-binding membrane-binding proteins present in all kingdoms of life (Rescher and Gerke, 2004). Plant annexins exhibit diverse activities, such as hydrolyzing ATP and GTP, forming Ca^2+^ channels, acting as peroxidases, or binding to F-actin (Mortimer et al., 2008). The cytokinin-regulated *ANNAT3* gene is also induced by H_2_O_2_ and ANNAT3 interacts with ANNAT1, a regulator of the H_2_O_2_-induced Ca^2+^ signal in Arabidopsis roots (Richards et al., 2014). ANNAT4 also interacts with ANNAT1 and plays a role in salt and drought stress adaptation (Huh et al., 2010).

Another remarkable cytokinin-induced gene is *AMP1*. The *amp1* mutant shows high cytokinin levels, altered embryonic patterning, faster vegetative growth, constitutive photomorphogenesis and precocious flowering (Chaudhury et al., 1993). The increased level of cytokinin corresponds with the de-etiolation response shown by *amp1* (Chin-Atkins et al., 1996) and is due to increased synthesis, not to decreased degradation, suggesting that AMP1 is a negative regulator of cytokinin synthesis (Nogué et al., 2000). *AMP1* encodes a putative carboxypeptidase with high similarity to N-acetyl α-linked acidic dipeptidases, suggesting that its product modulates the level of a small signaling molecule (Helliwell et al., 2001). *AMP1* interferes with auxin activity in meristem control, where *AMP1* acts as a differentiation-promoting agent (Vidaurre et al., 2007). However, the functional relevance of the mutual regulatory feedback between *AMP1* activity and cytokinin biosynthesis and its link to auxin action in regulating meristem activity awaits further analysis.

It is long known that the abundance of the *ACR4* transcript is increased in response to cytokinin (Hsieh and Goodman, 2002). The ACR domain, of which ACR4 (and the other plant ACR proteins) possess four repeats, serves in bacteria as an amino acid binding domain accomplishing allosteric regulation of the enzymatic activity of the protein in which it is located (Schuller et al., 1995; Grant et al., 2001). Unlike the bacterial proteins, plant ACR proteins have no known enzymatic domain and are probably pure receptors and/or signal transducers (Hsieh and Goodman, 2002). The only functional information comes from rice, where an ACR protein binds to a nuclear chaperone, but the meaning of this interaction is not understood (Hayakawa et al., 2006).

### Promoters of cytokinin-induced genes

Several investigations on the properties of cytokinin-regulated promoters have been made. Generally, it is an accepted model that type-B ARR proteins bind to specific *cis*-elements in the promoters of cytokinin-induced genes to activate their transcripton. It has been demonstrated that a pentanucleotide motif with the sequence AGAT[T,C] (cytokinin response motif, CRM) (Sakai et al., 2000; Hosoda et al., 2002; Imamura et al., 2003) is sufficient to bind type-B ARR proteins, and that ARR1 in particular binds to the octameric sequence AAGAT[T,C]TT (extended cytokinin response motif, ECRM) (Sakai et al., 2001; Taniguchi et al., 2007). The latter motif was shown to exhibit *cis*-regulatory activity *in planta* (Ramireddy et al., 2013).

Taking advantage of the very low probability of false positive genes, we investigated the occurrence, frequency, and distribution of these two motifs in the promoters of the core set of 65 cytokinin-induced genes from Table 2A (Supplementary Figure 2). Comparison with a set of 103 promoters of genes not regulated by cytokinin (listed in Supplementary Table 5) showed that the CRM is not overrepresented in cytokinin-responsive promoters. In contrast, the ECRM is significantly overrepresented about 2-fold in cytokinin-inducible promoters. However, it is missing in 36 of the 65 promoters (55%), among them those of several well-known immediate-early cytokinin response genes (e.g., *CKX4, CRF2, CRF5*, see Supplementary Figure 2). Consistent with earlier observations (Brenner et al., 2012), there must be other motifs that mediate cytokinin responsiveness. These could be other type-B response regulator binding sites, but also binding sites for the CRFs. Alternatively, there could be functionally relevant ECRMs outside the analyzed −1000 bp region, but these cases appear not to be very frequent (Franco-Zorrilla et al., 2014).

It could be that other type-B ARRs than ARR1 bind preferentially to different ECRM variants. It was indeed found that type-B response regulators belonging to different evolutionary lineages in Arabidopsis and rice have a differing capability to activate the Arabidopsis *ARR6* promoter (Kim et al., 2012; Ramireddy et al., 2013). On the other hand, our own bioinformatical analysis similar to that carried out to find the ECRM (Taniguchi et al., 2007) did not result in ECRM variants other than the known ARR1-binding AAGAT[T,C]TT motif (data not shown). In summary, we can conclude that neither is the presence of a certain frequency of CRMs diagnostic for a cytokinin-inducible promoter, nor does the absence of an ECRM exclude the cytokinin responsiveness of a promoter. Hence, a clear sequence motif-based definition of a cytokinin-inducible promoter is still missing.

**Figure 3:**
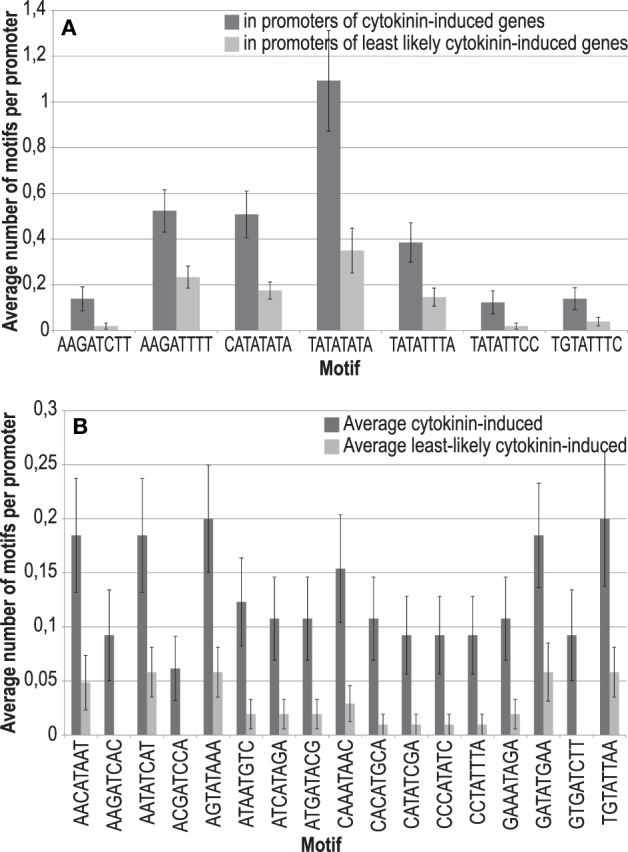
**Sequences and frequencies of novel potential type-B response regulator binding sites that were enriched in promoters of cytokinin-responsive genes in comparison to a control set of promoters. (A)** Enriched motifs derived from the consensus sequence (ACT)(AG)(GT)AT(ACT)(CT)(ACGT), characterized as binding motif for the type-B ARRs ARR11 and ARR14 (Franco-Zorrilla et al., 2014). **(B)** Enriched motifs derived from all possible DNA octamers containing a central AT.

Recently, highly degenerated novel binding motifs for the type-B ARRs ARR11 and ARR14 were identified using protein-binding microarrays containing oligonucleotide probes (Franco-Zorrilla et al., 2014). From these motifs we obtained the consensus sequence [ACT][AG][GT]AT[ACT][CT][ACGT]. We tested whether any single motif that can be derived from this consensus is enriched in cytokinin-responsive promoters. In addition to the ARR1 binding site, the ECRM AAGAT[TC]TT (Taniguchi et al., 2007; Bhargava et al., 2013), we found five more octameric sequence motifs enriched in cytokinin-responsive promoters: CATATATA, TATATATA, TATATTCC, TATATTTA, and TGTATTTC, albeit some with a *p*-value between 0.05 and 0.06 (Figure 3A, Supplementary Table 6).

To take this approach further, we reasoned that the central AT present in all motifs could be the only conserved sequence required for binding of this class of transcription factors. Therefore, we investigated whether any possible octamer containing a central AT is overrepresented in cytokinin-responsive promoters. In this analysis, we found 17 motifs, which are significantly overrepresented 2-fold or more (Figure 3B, Supplementary Table 7). Notably, the more frequent variant of the ARR1 binding motif (AAGATTTT) is not among the motifs that matched the criteria of a 2-fold enrichment for this most exhaustive analysis, having an enrichment of only 1.4-fold.

**Figure 4:**
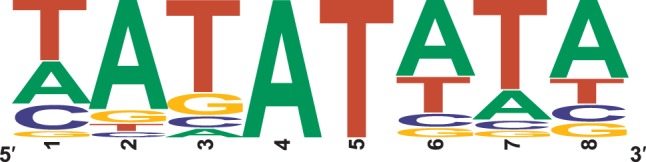
**Sequence logo of a novel model of a potential type-B response regulator binding site**. The motif combines all single motifs found in the analyses pictured in Figures 3A,B and shows the frequencies of the bases at the respective position in all promoters of the 65 cytokinin-regulated genes shown in Table 2A.

Combining the two analyses described before (Supplementary Tables 6, 7) revealed that all but one of the promoters of the advanced core set of cytokinin-responsive genes contains at least one of the motifs identified, and that, in total, these motifs are enriched almost 3.5-fold in comparison to a set of least likely cytokinin-regulated promoters (Supplementary Table 8). Figure 4 shows a sequence logo, which indicates the frequencies of the bases in the hypothetical cytokinin-responsive DNA element derived from this analysis. Evidently, this result is derived from a purely bioinformatical approach and needs experimental verification. However, this sequence model seems to be typical for type-B response regulators: All type-B response regulator binding sites characterized so far are AT-rich sequence motifs (Sakai et al., 2000, 2001; Hosoda et al., 2002; Imamura et al., 2003; Taniguchi et al., 2007; Franco-Zorrilla et al., 2014). By contrast, the typical sequence motifs for ERF/AP2 transcription factors, to which the CRFs belong, are GC-rich (Franco-Zorrilla et al., 2014).

Recently, an analysis of the *cis* elements bound by 24 different transcription families was published (Franco-Zorrilla et al., 2014). Motifs with similar properties as the novel candidate motifs proposed here, such as an AT in the center of the octamer, were found for MYB-related and MYB GARP-G2 transcription factors, both of which belong to the same superfamily of MYB transcription factors as the type-B response regulators, as well as for the C2C2 GATA-type protein GATA12. The flanking sequences of these motifs, however, differ from the ones of the candidate motifs proposed in Figure 3. Other transcription factor families binding to AT-rich sequence motifs are HD-ZIP proteins, C2C2 YABBY factors, and AHL proteins. The latter class binds to sequences containing no G or C at all. Apart from the motif ATATATAT for AHL12, none of the motifs identified by Franco-Zorrilla et al. (2014) is highly similar to the candidate motifs proposed in this work.

## Conclusion

Ten years of large-scale transcriptomics in the cytokinin field have yielded a sufficient amount of data to identify a core set of cytokinin-responsive genes. A large portion of the cytokinin-induced genes function in and around cytokinin signaling and metabolism. Of the remaining genes, there are several characterized examples encoding proteins with the potential of directly mediating cytokinin action, such as expansins, or the promoter of cell differentiation AMP1. The potential function of other genes, such as annexin genes, *ACR4*, or *ATR1*, in mediating cytokinin action is more enigmatic. Surprisingly, there are no consistently cytokinin-repressed genes. This may be partly due to the poor performance of microarrays in detecting low expression levels with little noise, which compromises reliable detection of downregulation.

One aspect of gene regulation that is missing from this analysis is the regulation of microRNAs. This important regulatory mechanism for decreasing gene activity is inevitably absent in this type of analysis because microRNAs are not represented on the microarrays considered here. The only information comes from the RNA sequencing analysis data (Bhargava et al., 2013), where among six non-protein coding genes two microRNAs were found to be regulated (miR163 and miR414), the former having a known target gene (AT1G15125, encoding a *S*-adenosyl-l-Met-dependent methyltransferase) whose transcript was consequently decreased. Unfortunately, the analysis presented here is lacking useful information on cytokinin-repressed transcripts, so a reverse search for potential microRNAs targeting these genes cannot be carried out.

Given the fact that in most cases the binding motifs for transcription factors are clearly and significantly overrepresented in their target promoters, the search for enriched motifs is an accepted way to identify new potential binding motifs in the promoters of a set of co-regulated genes. However, it was shown that the experimentally identified binding motifs of the type-B response regulators ARR11 and ARR14 are surprisingly not overrepresented in the promoters of cytokinin-regulated genes (Franco-Zorrilla et al., 2014), which may raise concerns whether this approach is appropriate in the case of cytokinin-regulated promoters. At this point, it should be noted that ARR11 and ARR14 are not considered to be the most important transcription factors for mediating cytokinin action. Thus, it may well be possible that the respective binding sites for the major type-B response regulators ARR1, ARR10, and ARR12 (Mason et al., 2005; Argyros et al., 2008; Ishida et al., 2008) may indeed be enriched in cytokinin-responsive promoters, and for the characterized binding motif of ARR1, the ECRM, this is a true fact (Supplementary Figure 2). For that reason, we are convinced that the overrepresentation analysis carried out here is a valid approach also in the case of the cytokinin signaling system. Our bioinformatic approaches to find alternative binding motifs using either degenerated motifs coming from an *in vitro* experiment or a randomly degenerated set of octamers has led to the identification of a number of enriched sequence motifs in cytokinin-responsive promoters. The AT-rich consensus of the motifs is consistent with the previously identified nature of type-B response regulator binding elements. As our analysis is pure bioinformatics, the true binding capacity of type-B response regulators to the novel candidate sequence motifs must ultimately be determined experimentally.

## Author contributions

Wolfram G. Brenner designed and carried out the research, evaluated the results, and contributed to writing the manuscript. Thomas Schmülling contributed to evaluating the results and writing the manuscript. Both authors finally approved the manuscript.

### Conflict of interest statement

The authors declare that the research was conducted in the absence of any commercial or financial relationships that could be construed as a potential conflict of interest.
