# Supplementary material for: Summarizing and exploring data of a decade of cytokinin-related transcriptomics
Source: Front Plant Sci. 2015 Feb 17;6:29. doi: 10.3389/fpls.2015.00029 (PMC4330702; doi:10.3389/fpls.2015.00029)
Supplement: Supplementary file 1 [file DataSheet1.PDF]

Supplementary Text. Program in Microsoft Visual Basic for applications (VBA) for counting motifs, exporting the results for each motif in each promoter in a tab-delimited text format, which can be easily imported into Microsoft Excel for further processing.

```
Sub ZaehleMotivePromotorweise()
'
' MotifCounting_Promoterwise Makro
'
'
    Selection.HomeKey unit:=wdStory
    MaxLaenge = 0
    Eingabedateiname = ActiveDocument.Name
    Eingabedateipfad = ActiveDocument.Path
    Dim Motivspeicher(10000)
    Dim Motivzaehler(10000)
    Dim Basenspeicher(20, 4)
    Dim Zaehler(20)
    AnzahlMotive = 0
    Eingabe = InputBox("Enter motif" + Chr$(13) + "Enclose degenerate bases in square" + Chr$(13) + "brackets and type them without separators.")
    ReverseComplement = MsgBox("Revers-komplementäre Motive mitzählen?", vbYesNo)
    Positionskorrektur = 0
    Degeneriert = False
    'Documents.Add
    For j = 1 To Len(Eingabe)
        If Mid$(Eingabe, j, 1) = "[" Then
           Degeneriert = True
           Positionskorrektur = Positionskorrektur - 1
           Ausgabeposition = -1
        ElseIf Mid$(Eingabe, j, 1) = "]" Then
           Degeneriert = False
           Positionskorrektur = Positionskorrektur + 1
        End If
        If Degeneriert Then
           Positionskorrektur = Positionskorrektur + 1
        Else
           Ausgabeposition = 0
        End If
        Ausgabeposition = Ausgabeposition + 1
        If Not (Mid$(Eingabe, j, 1) = "]" Or Mid$(Eingabe, j, 1) = "[") Then
           Basenspeicher(j - Positionskorrektur, Ausgabeposition) = Mid$(Eingabe, j, 1)
           Basenspeicher(j - Positionskorrektur, 0) = Right$(Str$(Ausgabeposition), 1)
'           Selection.TypeText Text:=Basenspeicher(j - Positionskorrektur, Ausgabeposition)
        End If
    Next
    Motivlaenge = Len(Eingabe) - Positionskorrektur
    Zaehlerzeiger = Motivlaenge
    For i = 1 To 20
        Zaehler(i) = 1
    Next
```

```
'    Stop
'    Documents.Add
    Do
       For i = 1 To Motivlaenge
           Motivspeicher(AnzahlMotive) = Motivspeicher(AnzahlMotive) + Basenspeicher(i, Zaehler(i))
'           Selection.TypeText Text:=Basenspeicher(i, Zaehler(i))
       Next
       AnzahlMotive = AnzahlMotive + 1
'       Selection.TypeParagraph
       zaehlende = False
       Do
          Zaehler(Zaehlerzeiger) = Zaehler(Zaehlerzeiger) + 1
          If Zaehler(Zaehlerzeiger) > CInt(Basenspeicher(Zaehlerzeiger, 0)) Then
             Zaehler(Zaehlerzeiger) = 1
             Zaehlerzeiger = Zaehlerzeiger - 1
             If Zaehlerzeiger = 0 Then
                fertig = True
                zaehlende = True
             End If
          Else
             Zaehlerzeiger = Motivlaenge
             zaehlende = True
          End If
       Loop Until zaehlende = True
    Loop Until fertig
'    Stop
'    ActiveDocument.Close
    If ReverseComplement = vbYes Then
       For i = 0 To AnzahlMotive - 1
           For j = Len(Motivspeicher(i)) To 1 Step -1
               Select Case Mid$(Motivspeicher(i), j, 1)
                      Case "A"
                           Motivspeicher(AnzahlMotive + i) = Motivspeicher(AnzahlMotive + i) + "T"
                      Case "C"
                           Motivspeicher(AnzahlMotive + i) = Motivspeicher(AnzahlMotive + i) + "G"
                      Case "G"
                           Motivspeicher(AnzahlMotive + i) = Motivspeicher(AnzahlMotive + i) + "C"
                      Case "T"
                           Motivspeicher(AnzahlMotive + i) = Motivspeicher(AnzahlMotive + i) + "A"
               End Select
           Next
       Next
    End If
    Documents.Add
    Selection.TypeText Text:="Gene name"
    For i = 0 To AnzahlMotive - 1
        Selection.TypeText Text:=vbTab + Motivspeicher(i)
    Next
    Selection.TypeParagraph
    ActiveDocument.SaveAs2 (Eingabedateipfad + "\" + Left$(Eingabedateiname, Len(Eingabedateiname) - 5) + "_Motif counts for" + Eingabe + ".docx")
```

```
    Ausgabedateiname = ActiveDocument.Name
    Documents(Eingabedateiname).Activate
    Selection.Find.ClearFormatting
    With Selection.Find
        .Text = ">"
        .Replacement.Text = ""
        .Forward = True
        .Wrap = wdFindStop
        .Format = False
        .MatchCase = False
        .MatchWholeWord = False
        .MatchWildcards = False
        .MatchSoundsLike = False
        .MatchAllWordForms = False
    End With
'    Stop
    MaxLaenge = Motivlaenge
    Do
        Found = Selection.Find.Execute
        If Found Then
           Selection.MoveRight unit:=wdCharacter, Count:=1
           Selection.MoveRight unit:=wdCharacter, Count:=9, Extend:=wdExtend
           Genname = Selection.Text
           Selection.EndKey unit:=wdLine
           Selection.MoveRight unit:=wdCharacter, Count:=1
           Do
              Selection.MoveRight unit:=wdCharacter, Count:=MaxLaenge, Extend:=wdExtend
              For i = 0 To AnzahlMotive - 1
                  If Left$(Selection.Text, Len(Motivspeicher(i))) = Motivspeicher(i) Then Motivzaehler(i) = Motivzaehler(i) + 1
                  If ReverseComplement = vbYes Then
                     If Not Motivspeicher(i) = Motivspeicher(i + AnzahlMotive) Then
                        If Left$(Selection.Text, Len(Motivspeicher(i))) = Motivspeicher(i + AnzahlMotive) Then Motivzaehler(i) = Motivzaehler(i) + 1
                     End If
                  End If
              Next
              Selection.MoveRight unit:=wdCharacter, Count:=1, Extend:=wdExtend
              If Right$(Selection.Text, 1) = Chr$(10) Or Right$(Selection.Text, 1) = Chr$(13) Or Len(Selection.Text) < MaxLaenge + 1 Then
promotorende = True Else promotorende = False
              Selection.MoveLeft unit:=wdCharacter, Count:=1
              Selection.MoveRight unit:=wdCharacter, Count:=1
           Loop Until promotorende
           Documents(Ausgabedateiname).Activate
           Selection.TypeText Text:=Genname
           For i = 0 To AnzahlMotive - 1
               Selection.TypeText Text:=vbTab + Right$(Str$(Motivzaehler(i)), 1)
           Next
           Selection.TypeParagraph
           Documents(Eingabedateiname).Activate
           For i = 0 To AnzahlMotive - 1
               Motivzaehler(i) = 0
```

```
            Next
        End If
    Loop Until Not Found
    Documents(Ausgabedateiname).Activate
    ActiveDocument.Save
    ActiveDocument.Close
    Beep
End Sub
```
