# Supplementary figures and images for: Summarizing and exploring data of a decade of cytokinin-related transcriptomics
Source: Front Plant Sci. 2015 Feb 17;6:29. doi: 10.3389/fpls.2015.00029 (PMC4330702; doi:10.3389/fpls.2015.00029)

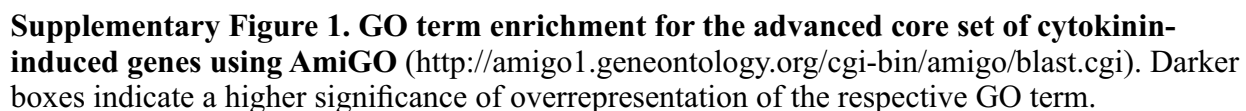

Supplement: Supplementary file 2 [file Image1.PDF]
