# Supplementary material for: Summarizing and exploring data of a decade of cytokinin-related transcriptomics
Source: Front Plant Sci. 2015 Feb 17;6:29. doi: 10.3389/fpls.2015.00029 (PMC4330702; doi:10.3389/fpls.2015.00029)
Supplement: Supplementary file 3 [file Image2.PDF]

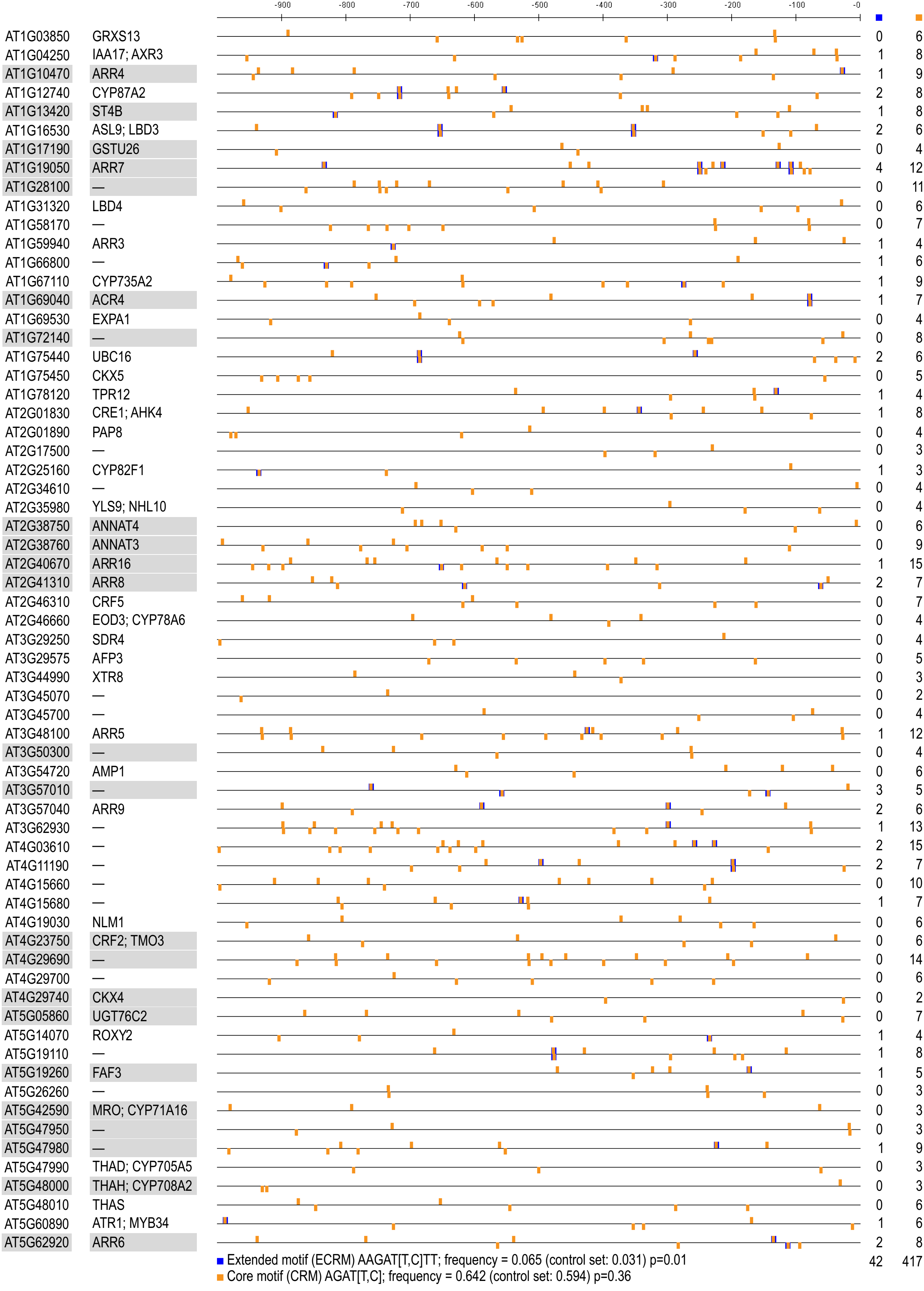

Supplementary Figure 2. Mapping of the two characterized cytokinin-responsive *cis*-elements CRM and ECRM to the promoters (–1000 bp regions) of the advanced core set of cytokinin-induced genes.
