# Supplementary material for: Summarizing and exploring data of a decade of cytokinin-related transcriptomics
Source: Front Plant Sci. 2015 Feb 17;6:29. doi: 10.3389/fpls.2015.00029 (PMC4330702; doi:10.3389/fpls.2015.00029)
Supplement: Supplementary file 6 [file Table3.PDF]

Supplementary Table 3. Cytokinin regulation of rice orthologs of the advanced core set of cytokinin-induced genes in Arabidopsis. This table is based on Table 2a. The comparison between Arabidopsis and rice is based on the evaluation of two publicly available rice microarray experiments containing cytokinin-induced samples downloaded from the GEO database under the accessions GSE6719 and GSE55902. The Arabidopsis genes listed here are induced by cytokinin treatment and found in at least three of four data sources (see Figure 1). Genes induced in all four data sources are marked with a grey background.

**AGI**	**Gene symbol**	**Description**	**Rice ortholog(s) assigned by WhETS [1] and by reciprocal BLAST**		**Regulated in rice dataset…**
			**TIGR ID**	**RAP ID**	
AT1G03850	*GRXS13*	glutaredoxin 13	LOC_Os01g47760<br>LOC_Os01g13950	Os01g0667900<br>Os01g0241400	no<br>no
AT1G04250	*IAA17; AXR3*	indole-3-acetic acid inducible 17; auxin resistant 3	LOC_Os01g08320<br>LOC_Os05g08570<br>LOC_Os06g39590<br>LOC_Os12g40890<br>LOC_Os03g43400	Os01g0178500<br>Os05g0178600<br>Os06g0597000<br>Os12g0601300<br>Os03g0633500	no<br>no<br>no<br>no<br>no
AT1G10470	*ARR4*	type-A response regulator 4	LOC_Os11g04720<br>no ID<br>LOC_Os01g72330	Os11g0143300<br>Os12g0139400<br>Os01g0952500	no<br>no<br>GSE6719 root 30, GSE6719 root 120, GSE6719 leaf 120, GSE55902
AT1G12740	*CYP87A2*	cytochrome P450, family 87, subfamily A, polypeptide 2	LOC_Os02g45280<br>LOC_Os03g45519<br>LOC_Os03g45619<br>LOC_Os04g48170<br>LOC_Os04g48200<br>LOC_Os04g48210<br>LOC_Os07g29960<br>LOC_Os10g10040<br>LOC_Os10g19110<br>LOC_Os10g19140<br>LOC_Os11g18570<br>LOC_Os12g18820<br>no ID	no ID<br>Os03g0658100<br>Os03g0658800<br>Os04g0570000<br>Os04g0570500<br>Os04g0570600<br>no ID<br>no ID<br>Os10g0337100<br>Os10g0337100<br>Os11g0289700<br>no ID<br>Os07g0482975	no<br>no<br>no<br>no<br>GSE6719 leaf 30<br>no<br>no<br>no<br>no<br>no<br>no<br>no<br>no
AT1G13420	*ST4B*	sulfotransferase 4B	LOC_Os07g05450<br>LOC_Os11g04540<br>LOC_Os04g44460<br>LOC_Os06g42120	no ID<br>Os12g0137900<br>Os04g0526300<br>Os06g0626600	no<br>no<br>no<br>no
AT1G16530	*ASL9; LBD3*	asymmetric leaves 2-like 9; LOB domain-containing protein 3	no rice ortholog		
AT1G17190	*GSTU26*	glutathione S-transferase tau 26	no rice ortholog		
AT1G19050	*ARR7*	type-A response regulator7	no rice ortholog		
AT1G28100		unknown protein	LOC_Os10g35644	Os10g0499600	no
AT1G31320	*LBD4*	LOB domain-containing protein 4	LOC_Os03g45750	Os03g0659700	no
AT1G58170		disease resistance-responsive (dirigent-like protein) family protein	no rice ortholog		no

AT1G59940	*ARR3*	type-A response regulator 3	LOC_Os02g42060<br>LOC_Os02g58350<br>LOC_Os04g36070<br>LOC_Os04g44280<br>LOC_Os07g26720<br>LOC_Os08g28900	no ID<br>Os02g0830200<br>Os04g0442300<br>Os04g0524300<br>no ID<br>no ID	no<br>no<br>GSE6719 root 120, GSE6719 leaf 120, GSE55902<br>no<br>no<br>no
AT1G66800		cinnamyl-alcohol dehydrogenase, putative	no rice ortholog		
AT1G67110	*CYP735A2*	cytochrome P450, family 735, subfamily A, polypeptide 2	LOC_Os06g09210<br>LOC_Os08g33300<br>LOC_Os09g23820	no ID<br>no ID<br>Os09g0403300	no<br>no<br>no
AT1G69040	*ACR4*	ACT domain repeat 4	LOC_Os03g29980<br>LOC_Os08g42100<br>LOC_Os03g40100<br>LOC_Os04g32110	Os03g0413100<br>Os08g0533600<br>Os03g0598100<br>Os04g0391500	no<br>GSE55902<br>no<br>no
AT1G69530	*EXPA1*	expansin A1	LOC_Os01g60770<br>LOC_Os05g39990<br>LOC_Os01g14650<br>LOC_Os02g51040	Os01g0823100<br>Os05g0477600<br>Os01g0248900<br>Os02g0744200	GSE6719 leaf 120<br>GSE6719 leaf 120<br>no<br>no
AT1G72140		major facilitator superfamily protein	LOC_Os01g65140	no ID	no
AT1G75440	*UBC16*	ubiquitin-conjugating enzyme 16	no rice ortholog		
AT1G75450	*CKX5*	cytokinin oxidase 5	LOC_Os01g10110<br>LOC_Os01g56810<br>LOC_Os06g37500	Os01g0197600<br>Os01g0775400<br>no ID	GSE6719 root 120, GSE6719 leaf 120<br>GSE6719 root 120<br>no
AT1G78120	*TPR12*	tetratricopeotide repeat 12	no rice ortholog		
AT2G01830	*CRE1; AHK4*	cytokinin response 1; arabidopsis histidine kinase 4	LOC_Os02g50480<br>LOC_Os03g50860<br>LOC_Os07g38910	no ID<br>Os03g0717700<br>no ID	no<br>no<br>GSE6719 leaf 120
AT2G01890	*PAP8*	purple acid phosphatase 8	LOC_Os03g13540<br>LOC_Os10g02750<br>LOC_Os11g34720	Os03g0238600<br>Os10g0116800<br>Os11g0549620	no<br>GSE6719 root 30, GSE6719 root 120<br>no
AT2G17500	*PILS5*	auxin efflux carrier family protein	LOC_Os09g38130<br>no ID	Os09g0554300<br>Os09g0555100	GSE6719 leaf 30<br>no
AT2G25160	*CYP82F1*	cytochrome P450, family 82, subfamily F, polypeptide 1	no rice ortholog		
AT2G34610		unknown protein	no rice ortholog		
AT2G35980	*YLS9; NHL10*	yellow-leaf-specific gene 9; NDR1/HIN1-like 10	LOC_Os01g64470<br>LOC_Os04g58850	Os01g0864500<br>Os04g0685300	no<br>no
AT2G38750	*ANNAT4*	annexin 4	LOC_Os05g31750	Os05g0382600	no
AT2G38760	*ANNAT3*	annexin 3	LOC_Os05g31760<br>LOC_Os07g46550	Os05g0382900<br>no ID	no<br>no
AT2G40670	*ARR16*	type-A response regulator 16	no rice ortholog		
AT2G41310	*ARR8*	type-A response regulator 8	LOC_Os08g26990<br>LOC_Os08g28950<br>LOC_Os11g04720	no ID<br>no ID<br>Os11g0143300	no<br>no<br>no
AT2G46310	*CRF5*	cytokinin response factor 5	LOC_Os05g25260	Os05g0316800	no

AT2G46660	*EOD3; CYP78A6*	enhancer of DA1-1 3	LOC_Os03g04190<br>LOC_Os08g43390<br>LOC_Os09g35940	Os03g0134566<br>Os08g0547300<br>Os09g0528700	no<br>GSE6719 leaf 120<br>no
AT3G29250	*SDR4*	short-chain dehydrogenase reductase 4	LOC_Os03g61740	Os03g0833100	no
AT3G29575	*AFP3*	ABI five binding protein 3	LOC_Os03g30570<br>LOC_Os07g41160	Os03g0419100<br>Os07g0602900	no<br>no
AT3G44990	*XTR8*	xyloglucan endo-transglucosylase-related 8	LOC_Os10g39840<br>LOC_Os03g01800	Os10g0545500<br>Os03g0108300	no<br>no
AT3G45070		P-loop containing nucleoside triphosphate hydrolases superfamily protein	LOC_Os01g20950<br>LOC_Os04g29000<br>LOC_Os08g20130<br>LOC_Os08g40380<br>LOC_Os09g38239<br>LOC_Os10g11270<br>LOC_Os11g04530<br>LOC_Os12g04320<br>LOC_Os11g30890<br>LOC_Os12g04350<br>LOC_Os12g04320	Os01g0311600<br>Os04g0359300<br>Os08g0297800<br>Os08g0515000<br>Os09g0555150<br>Os10g0190100<br>Os12g0137600<br>Os12g0137700<br>no ID<br>no ID<br>Os01g0311600	no<br>no<br>no<br>no<br>GSE6719 root 120<br>no<br>no<br>no<br>no<br>no<br>no
AT3G45700		major facilitator superfamily protein	LOC_Os05g27304	Os05g0338933	no
AT3G48100	*ARR5*	type-A response regulator 5	no rice ortholog		
AT3G50300		HXXXD-type acyl-transferase family protein	no rice ortholog		
AT3G54720	*AMP1*	altered meristem program 1	LOC_Os01g54010<br>LOC_Os03g57660	Os01g0743300<br>Os03g0790600	no<br>GSE6719 root 30
AT3G57010		Calcium-dependent phosphotriesterase superfamily protein	no rice ortholog		
AT3G57040	*ARR9*	type-A response regulator 9	LOC_Os01g72330<br>LOC_Os02g35180<br>LOC_Os07g26720<br>LOC_Os11g04720<br>LOC_Os12g04500<br>no ID	Os01g0952500<br>Os02g0557800<br>no ID<br>Os11g0143300<br>no ID<br>Os12g0139400	GSE6719 root 30, GSE6719 root 120, GSE6719 leaf 120, GSE55902<br>GSE6719 leaf 120<br>no<br>no<br>no<br>no
AT3G62930		thioredoxin superfamily protein	no rice ortholog		
AT4G03610		metallo-hydrolase/oxidoreductase superfamily protein	no rice ortholog		
AT4G11190		disease resistance-responsive (dirigent-like protein) family protein	no rice ortholog		
AT4G15660		thioredoxin superfamily protein	no rice ortholog		
AT4G15680		thioredoxin superfamily protein	LOC_Os01g27140<br>LOC_Os12g35330	Os01g0368900<br>Os12g0538600	no<br>no
AT4G19030	*NLM1*	NOD26-like intrinsic protein 1;1	LOC_Os02g13870<br>LOC_Os01g10600	Os02g0232900<br>Os01g0202800	GSE6719 leaf 30<br>no
AT4G23750	*CRF2; TMO3*	cytokinin response factor 2; target of monopteros 3	LOC_Os01g12440<br>LOC_Os06g06540	Os01g0224100<br>no ID	no<br>no
AT4G29690		alkaline-phosphatase-like family protein	LOC_Os01g10020	Os01g0196600	no
AT4G29700		alkaline-phosphatase-like family protein	LOC_Os01g10020	Os01g0196600	no
AT4G29740	*CKX4*	cytokinin oxidase 4	LOC_Os02g12770	no ID	no
AT5G05860	*UGT76C2*	UDP-glucosyl transferase 76C2, cytokinin N-glucosyltransferase	LOC_Os07g13800	Os07g0241700	no

AT5G14070	*ROXY2*	glutaredoxin ROXY2	LOC_Os02g30850	Os02g0512400	GSE6719 leaf 30
AT5G19110		Eukaryotic aspartyl protease family protein	no rice ortholog		
AT5G19260	*FAF3*	fantastic four 3	LOC_Os02g30850	Os05g0390300	no
AT5G26260		TRAF-like family protein	no rice ortholog		
AT5G42590	*MRO; CYP71A16*	marneral oxidase	LOC_Os06g43470<br>LOC_Os06g43510	Os06g0641900<br>Os06g0642500	no<br>GSE6719 leaf 30, GSE6719 leaf 120
AT5G47950		HXXXD-type acyl-transferase family protein	no rice ortholog		
AT5G47980		HXXXD-type acyl-transferase family protein	no rice ortholog		
AT5G47990	*THAD; CYP705A5*	thalian-diol desaturase	no rice ortholog		
AT5G48000	*THAH; CYP708A2*	thalianol hydroxylase	no rice ortholog		
AT5G48010	*THAS*	thalianol synthase	no rice ortholog		
AT5G60890	*ATR1; MYB34*	altered tryptophan regulation 1; MYB domain protein 34	no rice ortholog		
AT5G62920	*ARR6*	type-A response regulator 6	LOC_Os04g57720	Os04g0673300	GSE6719 root 30, GSE6719 leaf 120

1. Mitchell, R.A.C., et al., *Wheat Estimated Transcript Server (WhETS): a tool to provide best estimate of hexaploid wheat transcript sequence.* Nucleic Acids Research, 2007. **35**(suppl 2): p. W148-W151.
