# Supplementary material for: Summarizing and exploring data of a decade of cytokinin-related transcriptomics
Source: Front Plant Sci. 2015 Feb 17;6:29. doi: 10.3389/fpls.2015.00029 (PMC4330702; doi:10.3389/fpls.2015.00029)
Supplement: Supplementary file 7 [file Table4.PDF]

Supplementary Table 4. Results of the GO term enrichment analysis for the advanced core set of cytokinin-induced genes.

GO Term	P-value	Sample frequency	Background frequency	Genes
GO:0009735 response to cytokinin	1.35e-15	13/64 (20.3%)	136/30320 (0.4%)	ARR7 RR16 RR3 ARR4 WOL GRXS13 ST4B ACR4 RR5 ARR6 ARR9 AT4G11190 ARR3
GO:0009736 cytokinin-activated signaling pathway	1.62e-14	11/64 (17.2%)	83/30320 (0.3%)	ARR7 RR16 RR3 ARR4 WOL ST4B RR5 ARR6 ARR9 AT4G11190 ARR3
GO:0071368 cellular response to cytokinin stimulus	1.86e-14	11/64 (17.2%)	84/30320 (0.3%)	ARR7 RR16 RR3 ARR4 WOL ST4B RR5 ARR6 ARR9 AT4G11190 ARR3
GO:0009725 response to hormone	7.78e-08	20/64 (31.2%)	1662/30320 (5.5%)	ARR7 EXPA1 AXR3 ANNAT4 RR16 MYB34 AFP3 RR3 ARR4 WOL GRXS13 ST4B ACR4 RR5 ARR6 CKX5 ARR9 AT4G11190 YLS9 ARR3
GO:0009719 response to endogenous stimulus	6.70e-07	20/64 (31.2%)	1879/30320 (6.2%)	ARR7 EXPA1 AXR3 ANNAT4 RR16 MYB34 AFP3 RR3 ARR4 WOL GRXS13 ST4B ACR4 RR5 ARR6 CKX5 ARR9 AT4G11190 YLS9 ARR3
GO:0009755 hormone-mediated signaling pathway	6.96e-07	14/64 (21.9%)	795/30320 (2.6%)	ARR7 AXR3 RR16 AFP3 RR3 ARR4 WOL ST4B RR5 ARR6 ARR9 AT4G11190 YLS9 ARR3
GO:0032870 cellular response to hormone stimulus	1.29e-06	14/64 (21.9%)	834/30320 (2.8%)	ARR7 AXR3 RR16 AFP3 RR3 ARR4 WOL ST4B RR5 ARR6 ARR9 AT4G11190 YLS9 ARR3
GO:0071495 cellular response to endogenous stimulus	1.39e-06	14/64 (21.9%)	839/30320 (2.8%)	ARR7 AXR3 RR16 AFP3 RR3 ARR4 WOL ST4B RR5 ARR6 ARR9 AT4G11190 YLS9 ARR3
GO:0044699 single-organism process	1.69e-06	46/64 (71.9%)	10647/30320 (35.1%)	ARR7 EXPA1 NLM1 AXR3 AT3G57010 AT1G72140 RR16 MYB34 CYP708A2 AT3G62930 CYP87A2 AFP3 AT4G29700 RR3 ARR4 LBD4 AMP1 AT1G58170 PAP8 AT3G50300 AT1G66800 CYP705A5 AT2G17500 AT5G47980 WOL AT1G28100 AT3G45700 GRXS13 ST4B UBC16 GSTU26 CRF5 ACR4 RR5 ARR6 ROXY2 CKX5 CRF2 AT4G29690 ARR9 AT4G11190 THAS1 YLS9 ARR3 CYP78A6 UGT76C2
GO:0007623 circadian rhythm	2.91e-06	8/64 (12.5%)	177/30320 (0.6%)	ARR7 RR16 RR3 ARR4 RR5 ARR6 ARR9 ARR3
GO:0048511 rhythmic	2.91e-	8/64	177/30320	ARR7 RR16 RR3 ARR4 RR5 ARR6 ARR9 ARR3

process	06	(12.5%)	(0.6%)	
GO:0080003 thalianol metabolic process	6.77e-06	3/64 (4.7%)	3/30320 (0.0%)	CYP708A2 CYP705A5 THAS1
GO:0042221 response to chemical	1.11e-05	26/64 (40.6%)	3789/30320 (12.5%)	ARR7 EXPA1 NLM1 AXR3 ANNAT4 RR16 MYB34 CYP708A2 CYP87A2 AFP3 RR3 ARR4 PAP8 WOL GRXS13 ANNAT3 ST4B GSTU26 ACR4 RR5 ARR6 CKX5 ARR9 AT4G11190 YLS9 ARR3
GO:0010683 tricyclic triterpenoid metabolic process	2.70e-05	3/64 (4.7%)	4/30320 (0.0%)	CYP708A2 CYP705A5 THAS1
GO:0009812 flavonoid metabolic process	4.29e-05	8/64 (12.5%)	250/30320 (0.8%)	ARR7 RR3 ARR4 CYP705A5 WOL SULT202B1 RR5 UGT76C2
GO:0010033 response to organic substance	1.11e-04	21/64 (32.8%)	2805/30320 (9.3%)	ARR7 EXPA1 AXR3 ANNAT4 RR16 MYB34 AFP3 RR3 ARR4 WOL GRXS13 ST4B GSTU26 ACR4 RR5 ARR6 CKX5 ARR9 AT4G11190 YLS9 ARR3
GO:0071310 cellular response to organic substance	2.22e-04	14/64 (21.9%)	1261/30320 (4.2%)	ARR7 AXR3 RR16 AFP3 RR3 ARR4 WOL ST4B RR5 ARR6 ARR9 AT4G11190 YLS9 ARR3
GO:0065007 biological regulation	2.92e-04	29/64 (45.3%)	5427/30320 (17.9%)	ARR7 EXPA1 NLM1 AXR3 AT3G57010 RR16 MYB34 AFP3 CYP735A2 RR3 ARR4 LBD4 AMP1 AT2G17500 WOL ST4B CRF5 ACR4 RR5 ARR6 ROXY2 CKX5 CRF2 ARR9 AT4G11190 YLS9 CKX4 ARR3 UGT76C2
GO:0000160 phosphorelay signal transduction system	4.30e-04	6/64 (9.4%)	142/30320 (0.5%)	ARR7 RR3 WOL ARR6 ARR9 ARR3
GO:0007154 cell communication	6.85e-04	17/64 (26.6%)	2072/30320 (6.8%)	ARR7 AXR3 RR16 MYB34 CYP708A2 CYP87A2 AFP3 RR3 ARR4 WOL ST4B RR5 ARR6 ARR9 AT4G11190 YLS9 ARR3
GO:0031537 regulation of anthocyanin metabolic process	9.53e-04	4/64 (6.2%)	38/30320 (0.1%)	ARR7 RR3 WOL UGT76C2
GO:0070887 cellular response to chemical stimulus	1.13e-03	14/64 (21.9%)	1445/30320 (4.8%)	ARR7 AXR3 RR16 AFP3 RR3 ARR4 WOL ST4B RR5 ARR6 ARR9 AT4G11190 YLS9 ARR3
GO:0050896 response to stimulus	1.73e-03	31/64 (48.4%)	6618/30320 (21.8%)	ARR7 EXPA1 NLM1 AXR3 ANNAT4 AT1G72140 RR16 MYB34 CYP708A2 CYP87A2 AFP3 RR3 ARR4 AMP1 AT1G58170 PAP8 CYP705A5 WOL GRXS13 ANNAT3 ST4B UBC16 GSTU26 ACR4 RR5 ARR6 CKX5 ARR9 AT4G11190 YLS9 ARR3
GO:0044237 cellular	2.47e-	38/64	9492/30320	ARR7 XTH31 AT3G57010 TPR12 RR16 MYB34 CYP708A2 AFP3 AT4G29700 CYP735A2 RR3

metabolic process	03	(59.4%)	(31.3%)	ARR4 AT1G58170 PAP8 AT1G66800 CYP705A5 AT2G17500 WOL AT1G28100 AT3G45700 UBC16 GSTU26 CRF5 ASL9 ACR4 RR5 ARR6 ROXY2 CKX5 CRF2 AT4G29690 ARR9 AT4G11190 THAS1 YLS9 CKX4 ARR3 UGT76C2
GO:1901360 organic cyclic compound metabolic process	3.27e-03	26/64 (40.6%)	5035/30320 (16.6%)	ARR7 AT3G57010 RR16 MYB34 CYP708A2 AFP3 AT4G29700 CYP735A2 RR3 ARR4 AT1G58170 AT1G66800 CYP705A5 AT2G17500 AT1G28100 UBC16 CRF5 RR5 ARR6 ROXY2 CRF2 AT4G29690 ARR9 AT4G11190 THAS1 ARR3
GO:0007165 signal transduction	4.16e-03	14/64 (21.9%)	1617/30320 (5.3%)	ARR7 AXR3 RR16 AFP3 RR3 ARR4 WOL ST4B RR5 ARR6 ARR9 AT4G11190 YLS9 ARR3
GO:0008152 metabolic process	4.18e-03	40/64 (62.5%)	10544/30320 (34.8%)	ARR7 XTH31 AT3G57010 TPR12 RR16 MYB34 CYP708A2 AFP3 AT4G29700 CYP735A2 RR3 ARR4 AMP1 AT1G58170 PAP8 AT1G66800 CYP705A5 AT2G17500 WOL AT1G28100 AT3G45700 UBC16 GSTU26 CRF5 ASL9 SULT202B1 ACR4 RR5 ARR6 ROXY2 CKX5 CRF2 AT4G29690 ARR9 AT4G11190 THAS1 YLS9 CKX4 ARR3 UGT76C2
GO:0009987 cellular process	4.82e-03	43/64 (67.2%)	11952/30320 (39.4%)	ARR7 EXPA1 XTH31 AXR3 AT3G57010 TPR12 RR16 MYB34 CYP708A2 CYP87A2 AFP3 AT4G29700 CYP735A2 RR3 ARR4 AT1G58170 PAP8 AT1G66800 CYP705A5 AT2G17500 AT5G47980 WOL AT1G28100 AT3G45700 ST4B UBC16 GSTU26 CRF5 ASL9 ACR4 RR5 ARR6 ROXY2 CKX5 CRF2 AT4G29690 ARR9 AT4G11190 THAS1 YLS9 CKX4 ARR3 UGT76C2
GO:0006725 cellular aromatic compound metabolic process	5.01e-03	25/64 (39.1%)	4813/30320 (15.9%)	ARR7 RR16 MYB34 AFP3 AT4G29700 CYP735A2 RR3 ARR4 AT1G58170 AT1G66800 AT2G17500 AT1G28100 UBC16 CRF5 RR5 ARR6 ROXY2 CKX5 CRF2 AT4G29690 ARR9 AT4G11190 CKX4 ARR3 UGT76C2
GO:1901362 organic cyclic compound biosynthetic process	6.22e-03	20/64 (31.2%)	3288/30320 (10.8%)	ARR7 AT3G57010 RR16 MYB34 AFP3 CYP735A2 RR3 ARR4 AT1G58170 AT1G66800 AT1G28100 CRF5 RR5 ARR6 ROXY2 CRF2 ARR9 AT4G11190 THAS1 ARR3
GO:0009690 cytokinin metabolic process	7.39e-03	4/64 (6.2%)	63/30320 (0.2%)	CYP735A2 CKX5 CKX4 UGT76C2
GO:0009308 amine metabolic process	7.91e-03	6/64 (9.4%)	235/30320 (0.8%)	MYB34 CYP735A2 ACR4 CKX5 CKX4 UGT76C2
GO:0044700 single organism signaling	7.97e-03	14/64 (21.9%)	1712/30320 (5.6%)	ARR7 AXR3 RR16 AFP3 RR3 ARR4 WOL ST4B RR5 ARR6 ARR9 AT4G11190 YLS9 ARR3
GO:0023052 signaling	8.03e-03	14/64 (21.9%)	1713/30320 (5.6%)	ARR7 AXR3 RR16 AFP3 RR3 ARR4 WOL ST4B RR5 ARR6 ARR9 AT4G11190 YLS9 ARR3

GO Term	P-value	Sample frequency	Background frequency	Genes
GO:0000156 phosphorelay response regulator activity	4.25e-12	8/64 (12.5%)	35/30320 (0.1%)	ARR7 RR16 RR3 ARR4 RR5 ARR6 ARR9 ARR3
GO:0004871 signal transducer activity	8.16e-07	9/64 (14.1%)	224/30320 (0.7%)	ARR7 RR16 RR3 ARR4 WOL RR5 ARR6 ARR9 ARR3
GO:0060089 molecular transducer activity	8.16e-07	9/64 (14.1%)	224/30320 (0.7%)	ARR7 RR16 RR3 ARR4 WOL RR5 ARR6 ARR9 ARR3
GO:0019825 oxygen binding	4.54e-04	7/64 (10.9%)	232/30320 (0.8%)	CYP82F1 CYP71A16 CYP708A2 CYP87A2 CYP735A2 CYP705A5 CYP78A6
GO:0030613 oxidoreductase activity, acting on phosphorus or arsenic in donors	2.42e-03	3/64 (4.7%)	14/30320 (0.0%)	AT3G62930 AT4G15680 AT4G15660
GO:0030614 oxidoreductase activity, acting on phosphorus or arsenic in donors, disulfide as acceptor	2.42e-03	3/64 (4.7%)	14/30320 (0.0%)	AT3G62930 AT4G15680 AT4G15660
GO:0008794 arsenate reductase (glutaredoxin) activity	2.42e-03	3/64 (4.7%)	14/30320 (0.0%)	AT3G62930 AT4G15680 AT4G15660
GO:0030611 arsenate reductase activity	3.03e-03	3/64 (4.7%)	15/30320 (0.0%)	AT3G62930 AT4G15680 AT4G15660
