# Supplementary material for: Summarizing and exploring data of a decade of cytokinin-related transcriptomics
Source: Front Plant Sci. 2015 Feb 17;6:29. doi: 10.3389/fpls.2015.00029 (PMC4330702; doi:10.3389/fpls.2015.00029)
Supplement: Supplementary file 8 [file Table5.PDF]

Supplementary Table 5. Control set for the motif enrichment analysis consisting of the 103 genes with the lowest p-value for cytokinin induction from the meta-analysis of the CATMA microarray data

Locus Identifier	Gene Model Description	Primary Gene Symbol	All Gene Symbols
AT1G01340	member of Cyclic nucleotide gated channel family	CYCLIC NUCLEOTIDE GATED CHANNEL 10 (CNGC10)	CYCLIC NUCLEOTIDE GATED CHANNEL 10 (CNGC10)
AT1G02840	SR34/SR1 is a plant homologue of the human general/alternative splicing factor SF2/ASF. Barta et al (2010) have proposed a nomenclature for Serine/Arginine-Rich Protein Splicing Factors (SR proteins): Plant Cell. 2010, 22:2926.	SERINE/ARGININE-RICH PROTEIN SPLICING FACTOR 34 (SR34)	(SR1); (ATSRP34);SERINE/ARGININE-RICH PROTEIN SPLICING FACTOR 34 (At-SR34);SERINE/ARGININE-RICH PROTEIN SPLICING FACTOR 34 (SR34); (SRP34)
AT1G04120	encodes a high-affinity inositol hexakisphosphate transporter that plays a role in guard cell signaling and phytate storage. It is a member of MRP subfamily / ABC transporter subfamily C.	ATP-BINDING CASSETTE C5 (ABCC5)	MULTIDRUG RESISTANCE-ASSOCIATED PROTEIN 5 (ATMRP5);MULTIDRUG RESISTANCE-ASSOCIATED PROTEIN 5 (MRP5);ARABIDOPSIS THALIANA ATP-BINDING CASSETTE C5 (ATABCC5);ATP-BINDING CASSETTE C5 (ABCC5);MULTIDRUG RESISTANCE PROTEIN 5 (MRP5)
AT1G04410	predicted to encode a cytosolic malate dehydrogenase.	CYTOSOLIC-NAD-DEPENDENT MALATE DEHYDROGENASE 1 (c-NAD-MDH1)	CYTOSOLIC-NAD-DEPENDENT MALATE DEHYDROGENASE 1 (c-NAD-MDH1)
AT1G07830	ribosomal protein L29 family protein; FUNCTIONS IN: structural constituent of ribosome; INVOLVED IN: translation; LOCATED IN: mitochondrion, ribosome, mitochondrial ribosome, intracellular; EXPRESSED IN: 22 plant structures; EXPRESSED DURING: 13 growth stages; CONTAINS InterPro DOMAIN/s: Ribosomal protein L47, mitochondrial (InterPro:IPR010729), Ribosomal protein L29 (InterPro:IPR001854); Has 365 Blast hits to 365 proteins in 201 species: Archae - 0; Bacteria - 2; Metazoa - 115; Fungi - 146; Plants - 47; Viruses - 0; Other Eukaryotes - 55 (source: NCBI BLink).		
AT1G09810	evolutionarily conserved C-terminal region 11 (ECT11); CONTAINS InterPro DOMAIN/s: YTH domain (InterPro:IPR007275); BEST Arabidopsis thaliana protein match is: evolutionarily conserved C-terminal region 8 (TAIR:AT1G79270.1); Has 926 Blast hits to 916 proteins in 151 species: Archae - 0; Bacteria - 0; Metazoa - 416; Fungi - 98; Plants - 333; Viruses - 0; Other Eukaryotes - 79 (source: NCBI BLink).	EVOLUTIONARILY CONSERVED C-TERMINAL REGION 11 (ECT11)	EVOLUTIONARILY CONSERVED C-TERMINAL REGION 11 (ECT11)

AT1G13570	F-box/RNI-like superfamily protein; CONTAINS InterPro DOMAIN/s: F-box domain, cyclin-like (InterPro:IPR001810), FBD (InterPro:IPR013596), F-box domain, Skp2-like (InterPro:IPR022364), FBD-like (InterPro:IPR006566), Leucine-rich repeat 2 (InterPro:IPR013101); BEST Arabidopsis thaliana protein match is: F-box/RNI-like/FBD-like domains-containing protein (TAIR:AT5G56370.2); Has 1866 Blast hits to 1838 proteins in 25 species: Archae - 0; Bacteria - 0; Metazoa - 0; Fungi - 0; Plants - 1866; Viruses - 0; Other Eukaryotes - 0 (source: NCBI BLink).		
AT1G15200	protein-protein interaction regulator family protein; FUNCTIONS IN: molecular_function unknown; INVOLVED IN: biological_process unknown; LOCATED IN: cellular_component unknown; EXPRESSED IN: 22 plant structures; EXPRESSED DURING: 13 growth stages; CONTAINS InterPro DOMAIN/s: Pinin/SDK/memA protein (InterPro:IPR006786).		
AT1G19920	encodes a chloroplast form of ATP sulfurylase.	(APS2)	(APS2);ATP SULFURYLASE ARABIDOPSIS 1 (ASA1)
AT1G23260	MMZ1/UEV1A encodes a protein that may play a role in DNA damage responses and error-free post-replicative DNA repair by participating in lysine-63-based polyubiquitination reactions. UEV1A can form diubiquitin and triubiquitin chains in combination with UBC13A/UBC35 in vitro. It can also functionally complement an mms2 mutation in budding yeast, both by increasing mms2 mutant viability in the presence of the DNA damaging agent MMS, and by reducing the rate of spontaneous DNA mutation. However, a combination of MMZ1/UEV1A and UBC13A do not do a good job of rescuing an mms2 ubc13 double mutant in yeast. MMZ1/UEV1A transcripts are found at low levels in most plant organs, but cannot be detected in the pollen. Transcript levels do not appear to be stress-inducible. The uev1a-1 mutant shows normal sensitivity to MMS in germination assays suggesting that UEV1A is not required for DNA damage tolerance during this developmental stage.	MMS ZWEI HOMOLOGUE 1 (MMZ1)	MMS ZWEI HOMOLOGUE 1 (MMZ1);UBIQUITIN E2 VARIANT 1A (UEV1A)

AT1G32050	SCAMP family protein; FUNCTIONS IN: transmembrane transporter activity; INVOLVED IN: protein transport; LOCATED IN: mitochondrion, plasma membrane; EXPRESSED IN: 23 plant structures; EXPRESSED DURING: 14 growth stages; CONTAINS InterPro DOMAIN/s: SCAMP (InterPro:IPR007273); BEST Arabidopsis thaliana protein match is: secretory carrier 3 (TAIR:AT1G61250.1); Has 679 Blast hits to 677 proteins in 107 species: Archae - 0; Bacteria - 0; Metazoa - 375; Fungi - 14; Plants - 225; Viruses - 0; Other Eukaryotes - 65 (source: NCBI BLink).	SECRETORY CARRIER MEMBRANE PROTEIN 5 (SCAMP5)	SECRETORY CARRIER MEMBRANE PROTEIN 5 (SCAMP5); (ATSCAMP5)
AT1G32210	Encodes protein involved in suppression of apoptosis. Complements a mammalian apoptosis suppressor mutation.	DEFENDER AGAINST APOPTOTIC DEATH 1 (ATDAD1)	DEFENDER AGAINST APOPTOTIC DEATH 1 (ATDAD1)
AT1G32440	encodes a chloroplast pyruvate kinase beta subunit. The enzyme is less active than the other chloroplast pyruvate kinase beta subunit encoded by AT5G52920. Involved in seed oil biosynthesis. Can partially complement the AT5G52920 mutant.	PLASTIDIAL PYRUVATE KINASE 3 (PKp3)	PLASTIDIAL PYRUVATE KINASE 3 (PKp3)
AT1G33110	MATE efflux family protein; FUNCTIONS IN: antiporter activity, drug transmembrane transporter activity, transporter activity; INVOLVED IN: drug transmembrane transport, transmembrane transport; LOCATED IN: membrane; EXPRESSED IN: 22 plant structures; EXPRESSED DURING: 13 growth stages; CONTAINS InterPro DOMAIN/s: Multi antimicrobial extrusion protein MatE (InterPro:IPR002528); BEST Arabidopsis thaliana protein match is: MATE efflux family protein (TAIR:AT1G33100.1); Has 9952 Blast hits to 9876 proteins in 1988 species: Archae - 205; Bacteria - 7065; Metazoa - 140; Fungi - 326; Plants - 1371; Viruses - 0; Other Eukaryotes - 845 (source: NCBI BLink).		
AT1G33990	Encodes a protein predicted to act as a carboxylesterase. It has similarity to the SABP2 methyl salicylate esterase from tobacco. This protein does not act on methyl IAA, methyl JA, MeSA, MeGA4, or MEGA9 in vitro.	METHYL ESTERASE 14 (MES14)	METHYL ESTERASE 14 (MES14);METHYL ESTERASE 14 (ATMES14)

AT1G44760	Adenine nucleotide alpha hydrolases-like superfamily protein; FUNCTIONS IN: molecular_function unknown; INVOLVED IN: response to stress; EXPRESSED IN: 22 plant structures; EXPRESSED DURING: 13 growth stages; CONTAINS InterPro DOMAIN/s: UspA (InterPro:IPR006016), Rossmann-like alpha/beta/alpha sandwich fold (InterPro:IPR014729); BEST Arabidopsis thaliana protein match is: Adenine nucleotide alpha hydrolases-like superfamily protein (TAIR:AT1G69080.1); Has 216 Blast hits to 216 proteins in 22 species: Archae - 0; Bacteria - 0; Metazoa - 1; Fungi - 0; Plants - 214; Viruses - 0; Other Eukaryotes - 1 (source: NCBI BLink).		
AT1G45130	beta-galactosidase 5 (BGAL5); FUNCTIONS IN: cation binding, beta-galactosidase activity, hydrolase activity, hydrolyzing O-glycosyl compounds, catalytic activity; INVOLVED IN: lactose catabolic process, using glucoside 3-dehydrogenase, carbohydrate metabolic process, lactose catabolic process via UDP-galactose, lactose catabolic process; LOCATED IN: endomembrane system; EXPRESSED IN: 24 plant structures; EXPRESSED DURING: 13 growth stages; CONTAINS InterPro DOMAIN/s: Glycoside hydrolase, family 35, conserved site (InterPro:IPR019801), Glycoside hydrolase family 2, carbohydrate-binding (InterPro:IPR006104), Glycoside hydrolase, family 35 (InterPro:IPR001944), Glycoside hydrolase, catalytic core (InterPro:IPR017853), Glycoside hydrolase, subgroup, catalytic core (InterPro:IPR013781), Galactose-binding domain-like (InterPro:IPR008979); BEST Arabidopsis thaliana protein match is: beta-galactosidase 3 (TAIR:AT4G36360.1); Has 2206 Blast hits to 2062 proteins in 469 species: Archae - 15; Bacteria - 946; Metazoa - 364; Fungi - 218; Plants - 593; Viruses - 0; Other Eukaryotes - 70 (source: NCBI BLink).	BETA-GALACTOSIDASE 5 (BGAL5)	BETA-GALACTOSIDASE 5 (BGAL5)
AT1G47250	Encodes 20S proteasome subunit PAF2 (PAF2).	20S PROTEASOME ALPHA SUBUNIT F2 (PAF2)	20S PROTEASOME ALPHA SUBUNIT F2 (PAF2)
AT1G49480	Encodes a nuclear-localized DNA-binding protein that interacts with ITN1 at the PM and nuclei in vivo and may regulate ITN's subcellular localization.	RELATED TO VERNALIZATION1 1 (RTV1)	RELATED TO VERNALIZATION1 1 (RTV1)

AT1G62970	Chaperone DnaJ-domain superfamily protein; FUNCTIONS IN: heat shock protein binding; INVOLVED IN: protein folding; LOCATED IN: cellular_component unknown; EXPRESSED IN: 19 plant structures; EXPRESSED DURING: 9 growth stages; CONTAINS InterPro DOMAIN/s: Molecular chaperone, heat shock protein, Hsp40, DnaJ (InterPro:IPR015609), Heat shock protein DnaJ, N-terminal (InterPro:IPR001623); BEST Arabidopsis thaliana protein match is: Chaperone DnaJ-domain superfamily protein (TAIR:AT5G09540.1); Has 127172 Blast hits to 68298 proteins in 2468 species: Archae - 200; Bacteria - 18173; Metazoa - 48019; Fungi - 25520; Plants - 11802; Viruses - 2576; Other Eukaryotes - 20882 (source: NCBI BLink).		
AT1G63900	E3 Ubiquitin ligase family protein; FUNCTIONS IN: zinc ion binding; EXPRESSED IN: 22 plant structures; EXPRESSED DURING: 13 growth stages; CONTAINS InterPro DOMAIN/s: Zinc finger, RING-type (InterPro:IPR001841), E3 Ubiquitin ligase (InterPro:IPR022170); BEST Arabidopsis thaliana protein match is: E3 Ubiquitin ligase family protein (TAIR:AT1G59560.1).	DIAP1-LIKE PROTEIN 1 (DAL1)	DIAP1-LIKE PROTEIN 1 (DAL1)
AT1G64385	unknown protein; FUNCTIONS IN: molecular_function unknown; INVOLVED IN: biological_process unknown; LOCATED IN: endomembrane system; Has 66 Blast hits to 66 proteins in 21 species: Archae - 0; Bacteria - 0; Metazoa - 6; Fungi - 4; Plants - 51; Viruses - 0; Other Eukaryotes - 5 (source: NCBI BLink).		
AT1G68450	VQ motif-containing protein; FUNCTIONS IN: molecular_function unknown; INVOLVED IN: biological_process unknown; LOCATED IN: chloroplast; EXPRESSED IN: stem; CONTAINS InterPro DOMAIN/s: VQ (InterPro:IPR008889); BEST Arabidopsis thaliana protein match is: VQ motif-containing protein (TAIR:AT3G18360.1); Has 133 Blast hits to 133 proteins in 12 species: Archae - 0; Bacteria - 0; Metazoa - 0; Fungi - 0; Plants - 133; Viruses - 0; Other Eukaryotes - 0 (source: NCBI BLink).	PIGMENT DEFECTIVE 337 (PDE337)	PIGMENT DEFECTIVE 337 (PDE337)

AT1G68940	Armadillo/beta-catenin-like repeat family protein; FUNCTIONS IN: ubiquitin-protein ligase activity, binding; INVOLVED IN: protein ubiquitination; LOCATED IN: ubiquitin ligase complex; EXPRESSED IN: root; CONTAINS InterPro DOMAIN/s: U box domain (InterPro:IPR003613), Armadillo-like helical (InterPro:IPR011989), Armadillo (InterPro:IPR000225), Armadillo-type fold (InterPro:IPR016024); BEST Arabidopsis thaliana protein match is: senescence-associated E3 ubiquitin ligase 1 (TAIR:AT1G20780.1); Has 2072 Blast hits to 1938 proteins in 179 species: Archae - 0; Bacteria - 128; Metazoa - 110; Fungi - 40; Plants - 1633; Viruses - 3; Other Eukaryotes - 158 (source: NCBI BLink).		
AT1G70290	Encodes an enzyme putatively involved in trehalose biosynthesis. Though the protein has both trehalose-6-phosphate synthase (TPS)-like and trehalose-6-phosphate phosphatase (TPP)-like domains, neither activity has been detected in enzymatic assays nor has the protein been able to complement yeast TPS or TPP mutants.	TREHALOSE-6-PHOSPHATASE SYNTHASE S8 (TPS8)	(ATTPS8); (ATTPSC);TREHALOSE-6-PHOSPHATASE SYNTHASE S8 (TPS8)
AT1G70580	Encodes a protein with glyoxylate aminotransferase activity. It can act on a number of different small substrates and amino acids in vitro.	ALANINE-2-OXOGLUTARATE AMINOTRANSFERASE 2 (AOAT2)	GLUTAMATE:GLYOXYLATE AMINOTRANSFERASE 2 (GGT2);ALANINE-2-OXOGLUTARATE AMINOTRANSFERASE 2 (AOAT2)
AT1G72180	Leucine-rich receptor-like protein kinase family protein; FUNCTIONS IN: protein serine/threonine kinase activity, kinase activity, ATP binding; INVOLVED IN: transmembrane receptor protein tyrosine kinase signaling pathway, protein amino acid phosphorylation; LOCATED IN: cellular_component unknown; EXPRESSED IN: 22 plant structures; EXPRESSED DURING: 13 growth stages; CONTAINS InterPro DOMAIN/s: Protein kinase, catalytic domain (InterPro:IPR000719), Leucine-rich repeat-containing N-terminal domain, type 2 (InterPro:IPR013210), Leucine-rich repeat (InterPro:IPR001611), Serine/threonine-protein kinase-like domain (InterPro:IPR017442), Protein kinase-like domain (InterPro:IPR011009), Serine/threonine-protein kinase, active site (InterPro:IPR008271); BEST Arabidopsis thaliana protein match is: HAESA-like 1 (TAIR:AT1G28440.1); Has 222922 Blast hits to 143384 proteins in 4612 species: Archae - 143; Bacteria - 22250; Metazoa - 69718; Fungi - 11673; Plants - 90830; Viruses - 408; Other Eukaryotes - 27900 (source: NCBI BLink).		

AT1G73600	S-adenosyl-L-methionine-dependent methyltransferases superfamily protein; FUNCTIONS IN: methyltransferase activity, phosphoethanolamine N-methyltransferase activity; INVOLVED IN: metabolic process; EXPRESSED IN: guard cell; CONTAINS InterPro DOMAIN/s: Methyltransferase type 11 (InterPro:IPR013216); BEST Arabidopsis thaliana protein match is: S-adenosyl-L-methionine-dependent methyltransferases superfamily protein (TAIR:AT3G18000.1); Has 22304 Blast hits to 21648 proteins in 2625 species: Archae - 587; Bacteria - 16224; Metazoa - 297; Fungi - 950; Plants - 536; Viruses - 5; Other Eukaryotes - 3705 (source: NCBI BLink).		
AT1G73650	FUNCTIONS IN: oxidoreductase activity, acting on the CH-CH group of donors; INVOLVED IN: lipid metabolic process; LOCATED IN: plasma membrane, vacuole; EXPRESSED IN: 24 plant structures; EXPRESSED DURING: 13 growth stages; CONTAINS InterPro DOMAIN/s: 3-oxo-5-alpha-steroid 4-dehydrogenase, C-terminal (InterPro:IPR001104), Protein of unknown function DUF1295 (InterPro:IPR010721); BEST Arabidopsis thaliana protein match is: Protein of unknown function (DUF1295) (TAIR:AT1G18180.1); Has 35333 Blast hits to 34131 proteins in 2444 species: Archae - 798; Bacteria - 22429; Metazoa - 974; Fungi - 991; Plants - 531; Viruses - 0; Other Eukaryotes - 9610 (source: NCBI BLink).		
AT1G73720	Encodes SMU1, a protein involved in RNA splicing.	SUPPRESSORS OF MEC-8 AND UNC-52 1 (SMU1)	SUPPRESSORS OF MEC-8 AND UNC-52 1 (SMU1)

AT1G74010	Calcium-dependent phosphotriesterase superfamily protein; FUNCTIONS IN: strictosidine synthase activity; INVOLVED IN: biosynthetic process; LOCATED IN: plant-type cell wall; EXPRESSED IN: 11 plant structures; EXPRESSED DURING: L mature pollen stage, M germinated pollen stage, 4 anthesis, C globular stage, petal differentiation and expansion stage; CONTAINS InterPro DOMAIN/s: Strictosidine synthase, conserved region (InterPro:IPR018119), Strictosidine synthase (InterPro:IPR004141), Six-bladed beta-propeller, TolB-like (InterPro:IPR011042); BEST Arabidopsis thaliana protein match is: strictosidine synthase 2 (TAIR:AT1G74020.1); Has 953 Blast hits to 943 proteins in 185 species: Archae - 1; Bacteria - 180; Metazoa - 219; Fungi - 0; Plants - 462; Viruses - 0; Other Eukaryotes - 91 (source: NCBI BLink).		
AT1G74330	Protein kinase superfamily protein; FUNCTIONS IN: protein serine/threonine kinase activity, protein kinase activity, ATP binding; INVOLVED IN: protein amino acid phosphorylation, N-terminal protein myristoylation; LOCATED IN: cellular_component unknown; EXPRESSED IN: 23 plant structures; EXPRESSED DURING: 14 growth stages; CONTAINS InterPro DOMAIN/s: Protein kinase, ATP binding site (InterPro:IPR017441), Serine/threonine-protein kinase domain (InterPro:IPR002290), Serine/threonine-protein kinase-like domain (InterPro:IPR017442), Protein kinase-like domain (InterPro:IPR011009), Serine/threonine-protein kinase, active site (InterPro:IPR008271), Protein kinase, catalytic domain (InterPro:IPR000719), Tyrosine-protein kinase, catalytic domain (InterPro:IPR020635); BEST Arabidopsis thaliana protein match is: Protein kinase superfamily protein (TAIR:AT1G18670.1); Has 123005 Blast hits to 121589 proteins in 4349 species: Archae - 90; Bacteria - 13409; Metazoa - 46270; Fungi - 12908; Plants - 29866; Viruses - 453; Other Eukaryotes - 20009 (source: NCBI BLink).		
AT1G76980	BEST Arabidopsis thaliana protein match is: embryo defective 2170 (TAIR:AT1G21390.1); Has 65 Blast hits to 65 proteins in 23 species: Archae - 0; Bacteria - 8; Metazoa - 1; Fungi - 8; Plants - 40; Viruses - 3; Other Eukaryotes - 5 (source: NCBI BLink).		

AT2G01260	Protein of unknown function (DUF789); CONTAINS InterPro DOMAIN/s: Protein of unknown function DUF789 (InterPro:IPR008507); BEST Arabidopsis thaliana protein match is: Protein of unknown function (DUF789) (TAIR:AT1G15030.1); Has 265 Blast hits to 263 proteins in 17 species: Archae - 0; Bacteria - 0; Metazoa - 0; Fungi - 0; Plants - 263; Viruses - 0; Other Eukaryotes - 2 (source: NCBI BLink).		
AT2G17120	Induction of chitin-responsive genes by chitin treatment is not blocked in the mutant.	LYSM DOMAIN GPI-ANCHORED PROTEIN 2 PRECURSOR (LYM2)	LYSM DOMAIN GPI-ANCHORED PROTEIN 2 PRECURSOR (LYM2);CEBIP-LIKE 1 (CL-1)
AT2G17280	Phosphoglycerate mutase family protein; FUNCTIONS IN: catalytic activity; INVOLVED IN: metabolic process; LOCATED IN: cellular_component unknown; EXPRESSED IN: 24 plant structures; EXPRESSED DURING: 13 growth stages; CONTAINS InterPro DOMAIN/s: Histidine phosphatase superfamily, clade-1 (InterPro:IPR013078); BEST Arabidopsis thaliana protein match is: Phosphoglycerate mutase family protein (TAIR:AT5G64460.8); Has 682 Blast hits to 682 proteins in 146 species: Archae - 0; Bacteria - 77; Metazoa - 0; Fungi - 321; Plants - 168; Viruses - 0; Other Eukaryotes - 116 (source: NCBI BLink).		
AT2G26830	embryo defective 1187 (emb1187); CONTAINS InterPro DOMAIN/s: Choline/ethanolamine kinase (InterPro:IPR002573), Protein kinase-like domain (InterPro:IPR011009); BEST Arabidopsis thaliana protein match is: Protein kinase superfamily protein (TAIR:AT4G09760.1); Has 1627 Blast hits to 1577 proteins in 424 species: Archae - 0; Bacteria - 424; Metazoa - 442; Fungi - 246; Plants - 164; Viruses - 0; Other Eukaryotes - 351 (source: NCBI BLink).	EMBRYO DEFECTIVE 1187 (emb1187)	EMBRYO DEFECTIVE 1187 (emb1187)

AT2G36130	Cyclophilin-like peptidyl-prolyl cis-trans isomerase family protein; FUNCTIONS IN: peptidyl-prolyl cis-trans isomerase activity; INVOLVED IN: protein folding; LOCATED IN: cellular_component unknown; EXPRESSED IN: 24 plant structures; EXPRESSED DURING: 13 growth stages; CONTAINS InterPro DOMAIN/s: Cyclophilin-like (InterPro:IPR015891), Peptidyl-prolyl cis-trans isomerase, cyclophilin-type (InterPro:IPR002130), Peptidyl-prolyl cis-trans isomerase, cyclophilin-type, conserved site (InterPro:IPR020892); BEST Arabidopsis thaliana protein match is: cyclophilin71 (TAIR:AT3G44600.1); Has 17839 Blast hits to 17712 proteins in 2717 species: Archae - 108; Bacteria - 7579; Metazoa - 2864; Fungi - 1418; Plants - 1272; Viruses - 0; Other Eukaryotes - 4598 (source: NCBI BLink).		
AT2G38140	plastid-specific ribosomal protein 4 (PSRP4) mRNA, complete	PLASTID-SPECIFIC RIBOSOMAL PROTEIN 4 (PSRP4)	PLASTID-SPECIFIC RIBOSOMAL PROTEIN 4 (PSRP4)
AT2G38880	Encodes a transcription factor from the nuclear factor Y (NF-Y) family, AtNF-YB1. Confers drought tolerance.	"NUCLEAR FACTOR Y, SUBUNIT B1" (NF-YB1)	"NUCLEAR FACTOR Y, SUBUNIT B1" (NF-YB1)
AT2G43610	Chitinase family protein; FUNCTIONS IN: chitin binding, chitinase activity; INVOLVED IN: carbohydrate metabolic process, cell wall macromolecule catabolic process; LOCATED IN: plasma membrane; EXPRESSED IN: 15 plant structures; EXPRESSED DURING: 7 growth stages; CONTAINS InterPro DOMAIN/s: Chitin-binding, type 1, conserved site (InterPro:IPR018371), Glycoside hydrolase, family 19 (InterPro:IPR016283), Chitin-binding, type 1 (InterPro:IPR001002), Glycoside hydrolase, family 19, catalytic (InterPro:IPR000726); BEST Arabidopsis thaliana protein match is: Chitinase family protein (TAIR:AT2G43620.1); Has 3095 Blast hits to 2792 proteins in 589 species: Archae - 0; Bacteria - 708; Metazoa - 34; Fungi - 309; Plants - 1802; Viruses - 51; Other Eukaryotes - 191 (source: NCBI BLink).		
AT2G44100	GDP dissociation inhibitor involved in vesicular membrane traffic	GUANOSINE NUCLEOTIDE DIPHOSPHATE DISSOCIATION INHIBITOR 1 (GDI1)	GUANOSINE NUCLEOTIDE DIPHOSPHATE DISSOCIATION INHIBITOR 1 (ATGDI1);GUANOSINE NUCLEOTIDE DIPHOSPHATE DISSOCIATION INHIBITOR 1 (GDI1); (AT-GDI1)

AT2G44180	Encodes a MAP2 like methionine aminopeptidase. In MAP1A mutant background plants show an increased sensitivity to fumagillin resulting in defects in development. Phenotype is similar to RNAi lines which knock out all MAP2/MAP1 loci.	METHIONINE AMINOPEPTIDASE 2A (MAP2A)	METHIONINE AMINOPEPTIDASE 2A (MAP2A)
AT2G45470	FASCICLIN-like arabinogalactan protein 8 (FLA8); LOCATED IN: anchored to plasma membrane, apoplast, plasma membrane, anchored to membrane, plant-type cell wall; EXPRESSED IN: 24 plant structures; EXPRESSED DURING: 14 growth stages; CONTAINS InterPro DOMAIN/s: FAS1 domain (InterPro:IPR000782); BEST Arabidopsis thaliana protein match is: FASCICLIN-like arabinogalactan-protein 10 (TAIR:AT3G60900.1); Has 14155 Blast hits to 6880 proteins in 856 species: Archae - 79; Bacteria - 4506; Metazoa - 1333; Fungi - 788; Plants - 1999; Viruses - 868; Other Eukaryotes - 4582 (source: NCBI BLink).	FASCICLIN-LIKE ARABINOGALACTAN PROTEIN 8 (FLA8)	ARABINOGALACTAN PROTEIN 8 (AGP8);FASCICLIN-LIKE ARABINOGALACTAN PROTEIN 8 (FLA8)
AT2G45790	encodes a phosphomannomutase, involved in ascorbate biosynthesis	PHOSPHOMANNOMUTASE (PMM)	PHOSPHOMANNOMUTASE (ATPMM);PHOSPHOMANNOMUTASE (PMM)
AT2G47730	Encodes glutathione transferase belonging to the phi class of GSTs. Naming convention according to Wagner et al. (2002).	GLUTATHIONE S-TRANSFERASE PHI 8 (GSTF8)	ARABIDOPSIS THALIANA GLUTATHIONE S-TRANSFERASE PHI 8 (ATGSTF8);GLUTATHIONE S-TRANSFERASE PHI 8 (GSTF8); (GST6);GLUTATHIONE S-TRANSFERASE (CLASS PHI) 5 (ATGSTF5)
AT3G01590	Galactose mutarotase-like superfamily protein; FUNCTIONS IN: isomerase activity, carbohydrate binding, aldose 1-epimerase activity, catalytic activity; INVOLVED IN: galactose metabolic process, carbohydrate metabolic process; LOCATED IN: cellular_component unknown; EXPRESSED IN: 22 plant structures; EXPRESSED DURING: 13 growth stages; CONTAINS InterPro DOMAIN/s: Glycoside hydrolase-type carbohydrate-binding (InterPro:IPR011013), Aldose 1-epimerase (InterPro:IPR008183), Glycoside hydrolase-type carbohydrate-binding, subgroup (InterPro:IPR014718); BEST Arabidopsis thaliana protein match is: aldose 1-epimerase family protein (TAIR:AT5G14500.1); Has 1892 Blast hits to 1891 proteins in 757 species: Archae - 0; Bacteria - 1255; Metazoa - 39; Fungi - 135; Plants - 251; Viruses - 0; Other Eukaryotes - 212 (source: NCBI BLink).		
AT3G09880	Encodes B' regulatory subunit of PP2A (AtB'beta).	(ATB' BETA)	(ATB' BETA)

AT3G09900	RAB GTPase homolog E1E (RABE1e); FUNCTIONS IN: GTP binding; INVOLVED IN: protein transport, small GTPase mediated signal transduction; LOCATED IN: plasma membrane; EXPRESSED IN: 22 plant structures; EXPRESSED DURING: 13 growth stages; CONTAINS InterPro DOMAIN/s: Ras GTPase (InterPro:IPR001806), Small GTP-binding protein (InterPro:IPR005225), Small GTPase (InterPro:IPR020851), Ras (InterPro:IPR013753), Ras small GTPase, Rab type (InterPro:IPR003579); BEST Arabidopsis thaliana protein match is: RAB GTPase homolog 8C (TAIR:AT5G03520.1); Has 29798 Blast hits to 29728 proteins in 826 species: Archae - 21; Bacteria - 199; Metazoa - 15588; Fungi - 4257; Plants - 3488; Viruses - 20; Other Eukaryotes - 6225 (source: NCBI BLink).	RAB GTPASE HOMOLOG E1E (RABE1e)	(ATRAB8E);RAB GTPASE HOMOLOG E1E (ATRABE1E);RAB GTPASE HOMOLOG E1E (RABE1e)
AT3G13225	WW domain-containing protein; CONTAINS InterPro DOMAIN/s: WW/Rsp5/WWP (InterPro:IPR001202); Has 1723 Blast hits to 1069 proteins in 210 species: Archae - 2; Bacteria - 74; Metazoa - 564; Fungi - 222; Plants - 86; Viruses - 6; Other Eukaryotes - 769 (source: NCBI BLink).		
AT3G15060	RAB GTPase homolog A1G (RABA1g); FUNCTIONS IN: GTP binding; INVOLVED IN: response to cadmium ion; EXPRESSED IN: 24 plant structures; EXPRESSED DURING: 15 growth stages; CONTAINS InterPro DOMAIN/s: Ras GTPase (InterPro:IPR001806), Small GTP-binding protein (InterPro:IPR005225), Small GTPase (InterPro:IPR020851), Ras (InterPro:IPR013753), Ras small GTPase, Rab type (InterPro:IPR003579), Rab11-related (InterPro:IPR015595); BEST Arabidopsis thaliana protein match is: RAB GTPase homolog A1F (TAIR:AT5G60860.1); Has 27884 Blast hits to 27831 proteins in 751 species: Archae - 32; Bacteria - 137; Metazoa - 14654; Fungi - 4198; Plants - 2964; Viruses - 20; Other Eukaryotes - 5879 (source: NCBI BLink).	RAB GTPASE HOMOLOG A1G (RABA1g)	RAB GTPASE HOMOLOG A1G (RABA1g);RAB GTPASE HOMOLOG A1G (AtRABA1g)

AT3G19460	Reticulon family protein; FUNCTIONS IN: molecular_function unknown; INVOLVED IN: biological_process unknown; LOCATED IN: endoplasmic reticulum; EXPRESSED IN: 24 plant structures; EXPRESSED DURING: 15 growth stages; CONTAINS InterPro DOMAIN/s: Reticulon (InterPro:IPR003388); BEST Arabidopsis thaliana protein match is: Reticulon family protein (TAIR:AT2G15280.1); Has 985 Blast hits to 985 proteins in 72 species: Archae - 0; Bacteria - 0; Metazoa - 550; Fungi - 0; Plants - 426; Viruses - 0; Other Eukaryotes - 9 (source: NCBI BLink).		
AT3G27300	glucose-6-phosphate dehydrogenase 5 (G6PD5); FUNCTIONS IN: glucose-6-phosphate dehydrogenase activity; INVOLVED IN: response to cadmium ion, pentose-phosphate shunt, oxidative branch, glucose metabolic process; LOCATED IN: cytosol, chloroplast; EXPRESSED IN: 25 plant structures; EXPRESSED DURING: 14 growth stages; CONTAINS InterPro DOMAIN/s: Glucose-6-phosphate dehydrogenase, active site (InterPro:IPR019796), Glucose-6-phosphate dehydrogenase, C-terminal (InterPro:IPR022675), NAD(P)-binding domain (InterPro:IPR016040), Glucose-6-phosphate dehydrogenase (InterPro:IPR001282), Glucose-6-phosphate dehydrogenase, NAD-binding (InterPro:IPR022674); BEST Arabidopsis thaliana protein match is: glucose-6-phosphate dehydrogenase 6 (TAIR:AT5G40760.1); Has 8384 Blast hits to 8367 proteins in 2341 species: Archae - 0; Bacteria - 5762; Metazoa - 904; Fungi - 180; Plants - 377; Viruses - 4; Other Eukaryotes - 1157 (source: NCBI BLink).	GLUCOSE-6-PHOSPHATE DEHYDROGENASE 5 (G6PD5)	GLUCOSE-6-PHOSPHATE DEHYDROGENASE 5 (G6PD5)
AT3G29390	Nuclear localized K-homology containing protein that interacts with AS1.	RS2-INTERACTING KH PROTEIN (RIK)	RS2-INTERACTING KH PROTEIN (RIK)
AT3G44330	INVOLVED IN: protein processing; LOCATED IN: mitochondrion, endoplasmic reticulum, plasma membrane, vacuole; EXPRESSED IN: 25 plant structures; EXPRESSED DURING: 13 growth stages; CONTAINS InterPro DOMAIN/s: Nicalin (InterPro:IPR016574), EF-Hand 1, calcium-binding site (InterPro:IPR018247), Nicastrin (InterPro:IPR008710); Has 245 Blast hits to 243 proteins in 99 species: Archae - 6; Bacteria - 10; Metazoa - 139; Fungi - 0; Plants - 46; Viruses - 0; Other Eukaryotes - 44 (source: NCBI BLink).		
AT3G48690	Encodes a protein with carboxylesterase whose activity was tested using both pNA and 2,4-D-methyl.	(CXE12)	(CXE12);ARABIDOPSIS THALIANA CARBOXYESTERASE 12 (AtCXE12)

AT3G48890	putative progesterone-binding protein homolog (Atmp2) mRNA,	MEMBRANE-ASSOCIATED PROGESTERONE BINDING PROTEIN 3 (MAPR3)	(ATMP2);MEMBRANE-ASSOCIATED PROGESTERONE BINDING PROTEIN 3 (MAPR3);MEMBRANE STEROID BINDING PROTEIN 2 (MSBP2);ARABIDOPSIS THALIANA MEMBRANE-ASSOCIATED PROGESTERONE BINDING PROTEIN 3 (AtMAPR3)
AT3G49560	Mitochondrial import inner membrane translocase subunit Tim17/Tim22/Tim23 family protein; FUNCTIONS IN: protein transporter activity, P-P-bond-hydrolysis-driven protein transmembrane transporter activity; INVOLVED IN: protein transport; LOCATED IN: in 6 components; EXPRESSED IN: 23 plant structures; EXPRESSED DURING: 13 growth stages; CONTAINS InterPro DOMAIN/s: Sterile alpha motif, type 1 (InterPro:IPR021129), Mitochondrial inner membrane translocase complex, subunit Tim17/22 (InterPro:IPR003397); BEST Arabidopsis thaliana protein match is: Mitochondrial import inner membrane translocase subunit Tim17/Tim22/Tim23 family protein (TAIR:AT5G24650.1); Has 96 Blast hits to 94 proteins in 24 species: Archae - 0; Bacteria - 0; Metazoa - 4; Fungi - 0; Plants - 87; Viruses - 0; Other Eukaryotes - 5 (source: NCBI BLink).		
AT3G51660	Tautomerase/MIF superfamily protein; FUNCTIONS IN: molecular_function unknown; INVOLVED IN: inflammatory response, response to other organism; LOCATED IN: peroxisome; EXPRESSED IN: 20 plant structures; EXPRESSED DURING: 13 growth stages; CONTAINS InterPro DOMAIN/s: Tautomerase (InterPro:IPR014347), Macrophage migration inhibitory factor (InterPro:IPR001398); BEST Arabidopsis thaliana protein match is: Tautomerase/MIF superfamily protein (TAIR:AT5G01650.1); Has 396 Blast hits to 396 proteins in 86 species: Archae - 0; Bacteria - 25; Metazoa - 165; Fungi - 0; Plants - 140; Viruses - 0; Other Eukaryotes - 66 (source: NCBI BLink).		
AT3G55380	ubiquitin-conjugating enzyme 14 (UBC14); CONTAINS InterPro DOMAIN/s: Ubiquitin-conjugating enzyme/RWD-like (InterPro:IPR016135), Ubiquitin-conjugating enzyme, E2 (InterPro:IPR000608); BEST Arabidopsis thaliana protein match is: ubiquitin carrier protein 7 (TAIR:AT5G59300.1).	UBIQUITIN-CONJUGATING ENZYME 14 (UBC14)	UBIQUITIN-CONJUGATING ENZYME 14 (UBC14)

AT3G59280	mutant exhibited resistance to growth on media containing thaxtomin due to a difference in the rate of uptake of the toxin.We proposed that TXR1 is a component of, or regulator of, a dispensable transport mechanism.	THAXTOMIN A RESISTANT 1 (TXR1)	THAXTOMIN A RESISTANT 1 (TXR1)
AT4G01870	tolB protein-related; FUNCTIONS IN: molecular_function unknown; INVOLVED IN: response to cyclopentenone; LOCATED IN: membrane; EXPRESSED IN: 22 plant structures; EXPRESSED DURING: 13 growth stages; CONTAINS InterPro DOMAIN/s: WD40-like Beta Propeller (InterPro:IPR011659), Peptidase S9B, dipeptidylpeptidase IV N-terminal (InterPro:IPR002469), Six-bladed beta-propeller, TolB-like (InterPro:IPR011042); BEST Arabidopsis thaliana protein match is: DPP6 N-terminal domain-like protein (TAIR:AT1G21680.1); Has 6090 Blast hits to 4220 proteins in 1098 species: Archae - 42; Bacteria - 3414; Metazoa - 20; Fungi - 42; Plants - 120; Viruses - 0; Other Eukaryotes - 2452 (source: NCBI BLink).		
AT4G02195	Encodes a member of SYP4 Gene Family that is a plant ortholog of the Tlg2/syntaxin16 Qa-SNARE. Together with SYP43, it regulates the secretory and vacuolar transport pathways in the post-Golgi network and maintains the morphology of the Golgi apparatus and TGN and is required for extracellular resistance responses to a fungal pathogen.	SYNTAXIN OF PLANTS 42 (SYP42)	SYNTAXIN OF PLANTS 42 (SYP42); (ATTLG2B); (ATSYP42); (TLG2B)
AT4G02480	AAA-type ATPase family protein; FUNCTIONS IN: nucleoside-triphosphatase activity, ATPase activity, nucleotide binding, ATP binding; LOCATED IN: chloroplast; EXPRESSED IN: 24 plant structures; EXPRESSED DURING: 14 growth stages; CONTAINS InterPro DOMAIN/s: ATPase, AAA+ type, core (InterPro:IPR003593), ATPase, AAA-type, core (InterPro:IPR003959), SMAD/FHA domain (InterPro:IPR008984), ATPase, AAA-type, conserved site (InterPro:IPR003960); BEST Arabidopsis thaliana protein match is: AAA-type ATPase family protein (TAIR:AT1G02890.1); Has 36961 Blast hits to 32268 proteins in 3150 species: Archae - 1594; Bacteria - 13825; Metazoa - 4872; Fungi - 3636; Plants - 2887; Viruses - 35; Other Eukaryotes - 10112 (source: NCBI BLink).		

AT4G08310	FUNCTIONS IN: molecular_function unknown; INVOLVED IN: biological_process unknown; LOCATED IN: cellular_component unknown; EXPRESSED IN: 25 plant structures; EXPRESSED DURING: 13 growth stages; CONTAINS InterPro DOMAIN/s: Histone chaperone domain CHZ (InterPro:IPR019098); BEST Arabidopsis thaliana protein match is: unknown protein (TAIR:AT1G44780.2); Has 53711 Blast hits to 33687 proteins in 1618 species: Archae - 142; Bacteria - 4400; Metazoa - 24303; Fungi - 6688; Plants - 2484; Viruses - 449; Other Eukaryotes - 15245 (source: NCBI BLink).		
AT4G12570	Knock-out mutants showed accelerated senescence of leaves.	UBIQUITIN PROTEIN LIGASE 5 (UPL5)	UBIQUITIN PROTEIN LIGASE 5 (UPL5)
AT4G13720	Inosine triphosphate pyrophosphatase family protein; FUNCTIONS IN: hydrolase activity, pyrophosphatase activity; INVOLVED IN: biological_process unknown; EXPRESSED IN: 22 plant structures; EXPRESSED DURING: 13 growth stages; CONTAINS InterPro DOMAIN/s: Ham1-like protein (InterPro:IPR002637); Has 30201 Blast hits to 17322 proteins in 780 species: Archae - 12; Bacteria - 1396; Metazoa - 17338; Fungi - 3422; Plants - 5037; Viruses - 0; Other Eukaryotes - 2996 (source: NCBI BLink).		
AT4G14950	KMS1 encode a endoplasmic reticulum protein involved in the early secretory pathway.	KILLING ME SLOWLY 1 (KMS1)	KILLING ME SLOWLY 1 (KMS1)
AT4G15610	Uncharacterised protein family (UPF0497); CONTAINS InterPro DOMAIN/s: Uncharacterised protein family UPF0497, trans-membrane plant (InterPro:IPR006702), Uncharacterised protein family UPF0497, trans-membrane plant subgroup (InterPro:IPR006459); BEST Arabidopsis thaliana protein match is: Uncharacterised protein family (UPF0497) (TAIR:AT3G06390.1); Has 30201 Blast hits to 17322 proteins in 780 species: Archae - 12; Bacteria - 1396; Metazoa - 17338; Fungi - 3422; Plants - 5037; Viruses - 0; Other Eukaryotes - 2996 (source: NCBI BLink).		

AT4G18360	Aldolase-type TIM barrel family protein; FUNCTIONS IN: glycolate oxidase activity, oxidoreductase activity, FMN binding, catalytic activity; INVOLVED IN: oxidation reduction, metabolic process; LOCATED IN: peroxisome; EXPRESSED IN: 12 plant structures; EXPRESSED DURING: 4 anthesis, F mature embryo stage, petal differentiation and expansion stage, E expanded cotyledon stage, D bilateral stage; CONTAINS InterPro DOMAIN/s: Aldolase-type TIM barrel (InterPro:IPR013785), FMN-dependent alpha-hydroxy acid dehydrogenase, active site (InterPro:IPR008259), FMN-dependent dehydrogenase (InterPro:IPR000262), Alpha-hydroxy acid dehydrogenase, FMN-dependent (InterPro:IPR012133); BEST Arabidopsis thaliana protein match is: Aldolase-type TIM barrel family protein (TAIR:AT3G14420.2); Has 1807 Blast hits to 1807 proteins in 277 species: Archae - 0; Bacteria - 0; Metazoa - 736; Fungi - 347; Plants - 385; Viruses - 0; Other Eukaryotes - 339 (source: NCBI BLink).		
AT4G21160	ADP-ribosylation factor GTPase-activating protein containing zinc finger and C2 domains and a novel PI-3-P-binding protein region. Binds PI-3-P. Highest expression levels in flowering tissue, rosettes and roots. A member of ARF GAP domain (AGD), A thaliana has 15 members, grouped into four classes.	(ZAC)	(ZAC);ARF-GAP DOMAIN 12 (AGD12)
AT4G29390	Ribosomal protein S30 family protein; FUNCTIONS IN: structural constituent of ribosome; INVOLVED IN: translation; LOCATED IN: cytosolic small ribosomal subunit, ribosome, nucleolus; EXPRESSED IN: 23 plant structures; EXPRESSED DURING: 13 growth stages; CONTAINS InterPro DOMAIN/s: Ribosomal protein S30 (InterPro:IPR006846); BEST Arabidopsis thaliana protein match is: Ribosomal protein S30 family protein (TAIR:AT5G56670.1); Has 682 Blast hits to 682 proteins in 246 species: Archae - 2; Bacteria - 0; Metazoa - 304; Fungi - 135; Plants - 106; Viruses - 1; Other Eukaryotes - 134 (source: NCBI BLink).		
AT4G30140	Member of the GDSL lipase/esterase family of proteins that functions as cutinase. Expressed in pollen and at the zone of lateral root emergence.	CUTICLE DESTRUCTING FACTOR 1 (CDEF1)	CUTICLE DESTRUCTING FACTOR 1 (CDEF1)

AT4G30260	Integral membrane Yip1 family protein; FUNCTIONS IN: molecular_function unknown; INVOLVED IN: biological_process unknown; LOCATED IN: plasma membrane; EXPRESSED IN: callus; CONTAINS InterPro DOMAIN/s: Yip1 domain (InterPro:IPR006977); BEST Arabidopsis thaliana protein match is: Integral membrane Yip1 family protein (TAIR:AT2G18840.1); Has 30201 Blast hits to 17322 proteins in 780 species: Archae - 12; Bacteria - 1396; Metazoa - 17338; Fungi - 3422; Plants - 5037; Viruses - 0; Other Eukaryotes - 2996 (source: NCBI BLink).		
AT4G35080	high-affinity nickel-transport family protein; FUNCTIONS IN: nickel ion transmembrane transporter activity, metal ion binding; INVOLVED IN: nickel ion transport, metal ion transport, transmembrane transport; LOCATED IN: integral to membrane, chloroplast; CONTAINS InterPro DOMAIN/s: Nickel/cobalt transporter, high-affinity (InterPro:IPR011541); BEST Arabidopsis thaliana protein match is: high-affinity nickel-transport family protein (TAIR:AT2G16800.1).		
AT4G35570	Encodes a protein belonging to the subgroup of HMGB (high mobility group B) proteins that have a distinctive DNA-binding motif, the HMG-box domain. The motif confers non-sequence specific interaction with linear DNA and structure-specific binding to distorted DNA sites. The HMGB proteins are involved in the assembly of nucleoprotein complexes. Cannot be phosphorylated by CK2alpha.	HIGH MOBILITY GROUP B5 (HMGB5)	HIGH MOBILITY GROUP B5 (HMGB5); (NFD05); (HMGD);NUCLEOSOME/CHROMATIN ASSEMBLY FACTOR 5 (NFD5)
AT4G39080	Vacuolar proton ATPase subunit VHA-a isoform 3. Localized in the tonoplast.	VACUOLAR PROTON ATPASE A3 (VHA-A3)	VACUOLAR PROTON ATPASE A3 (VHA-A3)

AT5G08415	Radical SAM superfamily protein; FUNCTIONS IN: 4 iron, 4 sulfur cluster binding, lipoic acid synthase activity, iron-sulfur cluster binding, lipoate synthase activity, catalytic activity; INVOLVED IN: lipoic acid biosynthetic process, lipoate biosynthetic process, metabolic process; LOCATED IN: chloroplast; EXPRESSED IN: 24 plant structures; EXPRESSED DURING: 15 growth stages; CONTAINS InterPro DOMAIN/s: Aldolase-type TIM barrel (InterPro:IPR013785), Lipoate synthase (InterPro:IPR003698), Elongator protein 3/MiaB/NifB (InterPro:IPR006638), Radical SAM (InterPro:IPR007197); Has 30201 Blast hits to 17322 proteins in 780 species: Archae - 12; Bacteria - 1396; Metazoa - 17338; Fungi - 3422; Plants - 5037; Viruses - 0; Other Eukaryotes - 2996 (source: NCBI BLink).		
AT5G08570	Pyruvate kinase family protein; FUNCTIONS IN: pyruvate kinase activity, potassium ion binding, magnesium ion binding, catalytic activity; INVOLVED IN: glycolysis; LOCATED IN: cellular_component unknown; EXPRESSED IN: 22 plant structures; EXPRESSED DURING: 13 growth stages; CONTAINS InterPro DOMAIN/s: Pyruvate kinase, C-terminal-like (InterPro:IPR015795), Pyruvate kinase, active site (InterPro:IPR018209), Pyruvate kinase, beta-barrel-like (InterPro:IPR011037), Pyruvate kinase, alpha/beta (InterPro:IPR015794), Pyruvate/Phosphoenolpyruvate kinase, catalytic core (InterPro:IPR015813), Pyruvate kinase (InterPro:IPR001697), Pyruvate kinase, barrel (InterPro:IPR015793); BEST Arabidopsis thaliana protein match is: Pyruvate kinase family protein (TAIR:AT5G63680.1); Has 10268 Blast hits to 10160 proteins in 2717 species: Archae - 168; Bacteria - 6093; Metazoa - 541; Fungi - 219; Plants - 538; Viruses - 0; Other Eukaryotes - 2709 (source: NCBI BLink).		
AT5G09320	VPS9B; CONTAINS InterPro DOMAIN/s: Vacuolar sorting protein 9, subgroup (InterPro:IPR013995), Vacuolar sorting protein 9 (InterPro:IPR003123); BEST Arabidopsis thaliana protein match is: Vacuolar sorting protein 9 (VPS9) domain (TAIR:AT3G19770.1); Has 1393 Blast hits to 1376 proteins in 227 species: Archae - 0; Bacteria - 0; Metazoa - 508; Fungi - 217; Plants - 509; Viruses - 0; Other Eukaryotes - 159 (source: NCBI BLink).	(VPS9B)	(VPS9B)

AT5G09770	Ribosomal protein L17 family protein; FUNCTIONS IN: structural constituent of ribosome; INVOLVED IN: translation; LOCATED IN: ribosome, intracellular; EXPRESSED IN: 22 plant structures; EXPRESSED DURING: 13 growth stages; CONTAINS InterPro DOMAIN/s: Ribosomal protein L17 (InterPro:IPR000456); BEST Arabidopsis thaliana protein match is: Ribosomal protein L17 family protein (TAIR:AT5G64650.1); Has 30201 Blast hits to 17322 proteins in 780 species: Archae - 12; Bacteria - 1396; Metazoa - 17338; Fungi - 3422; Plants - 5037; Viruses - 0; Other Eukaryotes - 2996 (source: NCBI BLink).		
AT5G19440	similar to Eucalyptus gunnii alcohol dehydrogenase of unknown physiological function (GI:1143445), apple tree, PIR:T16995; NOT a cinnamyl-alcohol dehydrogenase		
AT5G19500	Encodes a putative amino acid transporter that localizes to the chloroplast inner envelope membrane.		
AT5G19550	Nitrogen metabolism. Major cytosolic isoenzyme controlling aspartate biosynthesis in the light.	ASPARTATE AMINOTRANSFERASE 2 (ASP2)	ASPARTATE AMINOTRANSFERASE 2 (AAT2);ASPARTATE AMINOTRANSFERASE 2 (ASP2)
AT5G20950	Glycosyl hydrolase family protein; FUNCTIONS IN: hydrolase activity, hydrolyzing O-glycosyl compounds; INVOLVED IN: carbohydrate metabolic process; LOCATED IN: cell wall, membrane, plant-type cell wall; EXPRESSED IN: 25 plant structures; EXPRESSED DURING: 13 growth stages; CONTAINS InterPro DOMAIN/s: Glycoside hydrolase, family 3, N-terminal (InterPro:IPR001764), Glycoside hydrolase, family 3, C-terminal (InterPro:IPR002772), Glycoside hydrolase, catalytic core (InterPro:IPR017853); BEST Arabidopsis thaliana protein match is: Glycosyl hydrolase family protein (TAIR:AT5G20940.1); Has 10079 Blast hits to 9261 proteins in 1444 species: Archae - 71; Bacteria - 6091; Metazoa - 14; Fungi - 1552; Plants - 486; Viruses - 0; Other Eukaryotes - 1865 (source: NCBI BLink).		
AT5G23740	Encodes a putative ribosomal protein S11 (RPS11-beta).	RIBOSOMAL PROTEIN S11-BETA (RPS11-BETA)	RIBOSOMAL PROTEIN S11-BETA (RPS11-BETA)

AT5G28830	calcium-binding EF hand family protein; FUNCTIONS IN: calcium ion binding; INVOLVED IN: biological_process unknown; LOCATED IN: cellular_component unknown; CONTAINS InterPro DOMAIN/s: Calcium-binding EF-hand (InterPro:IPR002048); BEST Arabidopsis thaliana protein match is: Calcium-binding EF-hand family protein (TAIR:AT4G38810.2); Has 259 Blast hits to 210 proteins in 27 species: Archae - 0; Bacteria - 0; Metazoa - 1; Fungi - 0; Plants - 241; Viruses - 0; Other Eukaryotes - 17 (source: NCBI BLink).		
AT5G43600	Encodes a protein with ureidoglycolate amidohydrolase activity in vitro. It is 27% identical and 43% similar to the E. coli allantoate amidohydrolase (AAH), but, in vitro assays with purified protein and allantoate as a substrate do not show any increase in ammonium concentration, indicating that there this enzyme has no AAH activity.	UREIDOGLYCOLATE AMIDOHYDROLASE (UAH)	UREIDOGLYCOLATE AMIDOHYDROLASE (UAH);ARABIDOPSIS THALIANA ALLANTOATE AMIDOHYDROLASE 2 (ATAAH-2)
AT5G43830	Aluminium induced protein with YGL and LRDR motifs; FUNCTIONS IN: molecular_function unknown; INVOLVED IN: biological_process unknown; LOCATED IN: cytosol, nucleus; EXPRESSED IN: 26 plant structures; EXPRESSED DURING: 15 growth stages; BEST Arabidopsis thaliana protein match is: Aluminium induced protein with YGL and LRDR motifs (TAIR:AT3G22850.1); Has 1807 Blast hits to 1807 proteins in 277 species: Archae - 0; Bacteria - 0; Metazoa - 736; Fungi - 347; Plants - 385; Viruses - 0; Other Eukaryotes - 339 (source: NCBI BLink).		
AT5G47660	Homeodomain-like superfamily protein; CONTAINS InterPro DOMAIN/s: SANT, DNA-binding (InterPro:IPR001005), MADF domain (InterPro:IPR006578), MYB-like (InterPro:IPR017877); BEST Arabidopsis thaliana protein match is: Duplicated homeodomain-like superfamily protein (TAIR:AT5G28300.1); Has 1807 Blast hits to 1807 proteins in 277 species: Archae - 0; Bacteria - 0; Metazoa - 736; Fungi - 347; Plants - 385; Viruses - 0; Other Eukaryotes - 339 (source: NCBI BLink).		

AT5G53650	unknown protein; FUNCTIONS IN: molecular_function unknown; INVOLVED IN: biological_process unknown; LOCATED IN: endomembrane system; EXPRESSED IN: 23 plant structures; EXPRESSED DURING: 15 growth stages; Has 30201 Blast hits to 17322 proteins in 780 species: Archae - 12; Bacteria - 1396; Metazoa - 17338; Fungi - 3422; Plants - 5037; Viruses - 0; Other Eukaryotes - 2996 (source: NCBI BLink).		
AT5G58380	Encodes a CBL-interacting protein kinase with similarity to SOS protein kinase.	SOS3-INTERACTING PROTEIN 1 (SIP1)	CBL-INTERACTING PROTEIN KINASE 10 (CIPK10);SNF1-RELATED PROTEIN KINASE 3.8 (SNRK3.8);SOS3-INTERACTING PROTEIN 1 (SIP1); (PKS2)
AT5G59090	subtilase 4.12 (SBT4.12); FUNCTIONS IN: identical protein binding, serine-type endopeptidase activity; INVOLVED IN: proteolysis, negative regulation of catalytic activity; LOCATED IN: apoplast, nucleus, cytoplasm; EXPRESSED IN: 6 plant structures; CONTAINS InterPro DOMAIN/s: Protease-associated PA (InterPro:IPR003137), Peptidase S8/S53, subtilisin/kexin/sedolisin (InterPro:IPR000209), Peptidase S8, subtilisin-related (InterPro:IPR015500), Peptidase S8/S53, subtilisin, active site (InterPro:IPR022398), Proteinase inhibitor I9, subtilisin propeptide (InterPro:IPR010259); BEST Arabidopsis thaliana protein match is: subtilase 4.13 (TAIR:AT5G59120.1); Has 30201 Blast hits to 17322 proteins in 780 species: Archae - 12; Bacteria - 1396; Metazoa - 17338; Fungi - 3422; Plants - 5037; Viruses - 0; Other Eukaryotes - 2996 (source: NCBI BLink).	SUBTILASE 4.12 (SBT4.12)	SUBTILASE 4.12 (SBT4.12);SUBTILASE 4.12 (ATSBT4.12)
AT5G59480	Haloacid dehalogenase-like hydrolase (HAD) superfamily protein; FUNCTIONS IN: hydrolase activity; LOCATED IN: cellular_component unknown; EXPRESSED IN: 24 plant structures; EXPRESSED DURING: 15 growth stages; CONTAINS InterPro DOMAIN/s: Pyrimidine 5-nucleotidase (InterPro:IPR010237), HAD-superfamily hydrolase, subfamily IA, variant 3 (InterPro:IPR006402); BEST Arabidopsis thaliana protein match is: Haloacid dehalogenase-like hydrolase (HAD) superfamily protein (TAIR:AT5G02230.2); Has 2253 Blast hits to 2250 proteins in 467 species: Archae - 3; Bacteria - 705; Metazoa - 0; Fungi - 141; Plants - 199; Viruses - 0; Other Eukaryotes - 1205 (source: NCBI BLink).		

AT5G59540	2-oxoglutarate (2OG) and Fe(II)-dependent oxygenase superfamily protein; FUNCTIONS IN: oxidoreductase activity; INVOLVED IN: biological_process unknown; LOCATED IN: cellular_component unknown; EXPRESSED IN: 17 plant structures; EXPRESSED DURING: 9 growth stages; CONTAINS InterPro DOMAIN/s: Oxoglutarate/iron-dependent oxygenase (InterPro:IPR005123); BEST Arabidopsis thaliana protein match is: 2-oxoglutarate (2OG) and Fe(II)-dependent oxygenase superfamily protein (TAIR:AT5G59530.1); Has 1807 Blast hits to 1807 proteins in 277 species: Archae - 0; Bacteria - 0; Metazoa - 736; Fungi - 347; Plants - 385; Viruses - 0; Other Eukaryotes - 339 (source: NCBI BLink).		
AT5G61060	Encodes a member of the histone deacetylase family.	HISTONE DEACETYLASE 5 (HDA05)	HISTONE DEACETYLASE 5 (HDA05)
AT5G63510	Encodes a mitochondrial gamma carbonic anhydrase-like protein. Component of the NADH dehydrogenase complex.	GAMMA CARBONIC ANHYDRASE LIKE 1 (GAMMA CAL1)	GAMMA CARBONIC ANHYDRASE LIKE 1 (GAMMA CAL1)
AT5G63870	Encodes a nuclear localized serine/threonine phosphatase that appears to be regulated by redox activity and is a positive regulator of cryptochrome mediated blue light signalling.	SERINE/THREONINE PHOSPHATASE 7 (PP7)	SERINE/THREONINE PHOSPHATASE 7 (PP7)
AT5G64220	Calmodulin-binding transcription activator protein with CG-1 and Ankyrin domains; FUNCTIONS IN: calmodulin binding, transcription regulator activity; INVOLVED IN: regulation of transcription; LOCATED IN: nucleus; EXPRESSED IN: 23 plant structures; EXPRESSED DURING: 13 growth stages; CONTAINS InterPro DOMAIN/s: Ankyrin repeat-containing domain (InterPro:IPR020683), CG-1 (InterPro:IPR005559), IQ calmodulin-binding region (InterPro:IPR000048), Ankyrin repeat (InterPro:IPR002110); BEST Arabidopsis thaliana protein match is: ethylene induced calmodulin binding protein (TAIR:AT5G09410.2); Has 14517 Blast hits to 9081 proteins in 522 species: Archae - 46; Bacteria - 904; Metazoa - 8296; Fungi - 946; Plants - 937; Viruses - 67; Other Eukaryotes - 3321 (source: NCBI BLink).		

AT5G64240	Encodes a type I metacaspase. Two Arabidopsis metacaspases, AT1G02170 (MC1) and AT4G25110 (MC2) antagonistically control programmed cell death in Arabidopsis. MC1 is a positive regulator of cell death and requires conserved caspase-like putative catalytic residues for its function. MC2 negatively regulates cell death. This function is independent of the putative catalytic residues. A third type I Arabidopsis metacaspase is MC3 (AT5g64240).	METACASPASE 3 (MC3)	METACASPASE 3 (AtMC3);METACASPASE 1A (AtMCP1a);METACASPASE 1A (MCP1a);METACASPASE 3 (MC3)
AT5G64480	unknown protein; Has 35333 Blast hits to 34131 proteins in 2444 species: Archae - 798; Bacteria - 22429; Metazoa - 974; Fungi - 991; Plants - 531; Viruses - 0; Other Eukaryotes - 9610 (source: NCBI BLink).		
AT5G66490	unknown protein; FUNCTIONS IN: molecular_function unknown; INVOLVED IN: biological_process unknown; LOCATED IN: plasma membrane; EXPRESSED IN: 17 plant structures; EXPRESSED DURING: 9 growth stages; BEST Arabidopsis thaliana protein match is: unknown protein (TAIR:AT3G50900.1); Has 1807 Blast hits to 1807 proteins in 277 species: Archae - 0; Bacteria - 0; Metazoa - 736; Fungi - 347; Plants - 385; Viruses - 0; Other Eukaryotes - 339 (source: NCBI BLink).		
AT5G66870	Encodes LOB domain protein whose overexpression results in KNOX gene repression. Overexpression also results in plants with hyponastic leaves, downward pointing flowers and reduced apical dominance. May be involved in the transcriptional regulation of the homeobox gene BP (brevipedicellus) during lateral organ differentiation. Acts together with AS2 in proximal-distal symmetry determination.	ASYMMETRIC LEAVES 2-LIKE 1 (ASL1)	ASYMMETRIC LEAVES 2-LIKE 1 (ASL1);LATERAL ORGAN BOUNDARIES DOMAIN GENE 36 (LBD36)
