# Supplementary material for: Summarizing and exploring data of a decade of cytokinin-related transcriptomics
Source: Front Plant Sci. 2015 Feb 17;6:29. doi: 10.3389/fpls.2015.00029 (PMC4330702; doi:10.3389/fpls.2015.00029)
Supplement: Supplementary file 13 [file Table10.PDF]

Supplemental Table 10. Literature analysis of genes found regulated by cytokinin in different publications. The publications referenced in the table as numbers are listed after the end of the table.

AGI	Examples of regulated genes	References	No. of hits
AT4G29740	CKX4	2 3 4 6 8 9 10 11 12 13	10
AT1G69530	EXP1	1 2 3 5 6 8 9 13	8
AT4G23750	CRF2	1 3 4 6 7 8 9 12	8
AT1G17190	GSTU26	4 6 7 8 11 12 13	7
AT1G67110	CYP735A2	3 6 8 9 10 11 13	7
AT2G17820	AHK1	1 2 4 6 7 8 10	7
AT2G38750	ANNAT4	3 4 6 7 8 13	6
AT2G46310	CRF5	3 4 6 7 8 9	6
AT4G11190	Dirigent-like	3 4 6 8 9 11	6
AT4G19030	NLM1	3 6 7 8 9 11	6
AT1G16530	ASL9	3 6 8 10 11	5
AT2G30540	Thioredoxin	3 6 8 9 10	5
AT3G13790	Cell wall invertase 1	1 7 8 12 13	5
AT5G48000	THAH	6 7 8 12 13	5
AT5G60890	ATR1	1 2 6 8 12	5
AT1G04240	IAA3	3 5 7 9	4
AT1G04250	AXR3	1 3 8 12	4
AT1G58170	Dirigent-like	6 8 10 13	4
AT1G68360	Zinc finger superfam.	8 10 12 13	4
AT2G35980	YLS9	3 7 8 11	4
AT3G62930	Thioredoxin	4 6 8 9	4
AT4G11210	Dirigent-like	3 6 8 9	4
AT4G34590	BZIP11	1 2 5 13	4
AT4G39070	B-box type zinc finger	2 8 12 13	4
AT5G05860	UGT76C2	4 6 8 12	4
AT5G47990	THAD	4 6 7 8	4
AT1G13740	AFP2	5 8 13	3
AT1G14960	Llipid transport superfam.	6 7 8	3
AT1G15550	GA4	5 6 12	3
AT1G35140	EXL1	8 12 13	3
AT1G43160	RAP2.6	4 8 10	3
AT1G69040	ACR4	4 6 12	3
AT1G71030	MYBL2	3 5 13	3
AT1G75450	CKX5	2 5 8	3
AT1G77330	ACC oxidase-like	5 7 8	3
AT1G78000	SULTR1;2	5 8 13	3
AT1G78380	GST8	1 2 13	3
AT2G03090	EXP15	6 8 10	3
AT2G17500	Auxin efflux carrier fam.	7 12 13	3
AT2G21650	MEE3	3 6 8	3
AT2G29490	GST19	3 8 9	3
AT2G29500	HSP20-like	8 11 13	3
AT2G33830	Dormancy / auxin-associated fam.	5 9 13	3
AT2G35270	GIK	3 8 13	3
AT2G35940	BLH1	1 2 13	3
AT2G37040	PAL1	2 5 13	3
AT2G38760	ANN3	6 8 13	3
AT2G39220	PLP6	3 6 8	3
AT2G39705	DVL11	5 8 11	3
AT2G45220	Pectin methylesterase inhibitor superfam.	3 8 13	3
AT3G01190	Peroxidase	8 12 13	3
AT3G15990	SULTR3;4	8 12 13	3
AT3G16810	PUM24	4 6 13	3
AT3G44750	HD2A	4 6 13	3
AT3G48920	MYB45	2 8 10	3
AT3G57660	NRPA1	3 4 6	3
AT3G59480	pfkB-like carbohydrate kinase fam.	5 8 13	3
AT4G01250	WRKY22	2 12 13	3
AT4G01630	EXPA17	5 8 11	3

AGI	Examples of regulated genes	References	No. of hits
AT4G11280	ACS6	3 12 13	3
AT4G16990	RLM3	3 11 13	3
AT4G26150	GNL	5 6 8	3
AT4G27410	RD26	5 7 11	3
AT4G28270	RMA2	3 7 13	3
AT5G01530	LHCB4.1	1 2 13	3
AT5G12030	HSP17.6	3 7 11	3
AT5G15130	WRKY72	2 5 12	3
AT5G15960	KIN1	1 2 3	3
AT5G26220	ChaC-like fam.	7 8 11	3
AT5G39610	NAC2	11 12 13	3
AT5G47220	ERF2	3 12 13	3
AT5G48010	THAS	6 7 8	3
AT5G51440	HSP20-like	4 6 11	3
AT5G56320	EXP14	8 10 12	3
AT5G56970	CKX3	2 8 10	3
AT5G63160	BT1	7 12 13	3
AT5G64620	C/VIF2	3 6 13	3
AT5G67520	APK4	8 12 13	3
AT1G03850	Glutaredoxin	5 8	2
AT1G05650	Pectin lyase-like	5 8	2
AT1G05680	UGT74E2	7 11	2
AT1G09540	MYB61	3 8	2
AT1G10070	BCAT-2	5 13	2
AT1G10585	bHLH fam.	4 11	2
AT1G12110	NRT1.1	8 13	2
AT1G13300	HRS1	5 8	2
AT1G19180	JAZ1	12 13	2
AT1G19770	PUP14	12 13	2
AT1G20190	EXP11	6 13	2
AT1G20620	SEN2	1 2	2
AT1G21910	DREB26	12 13	2
AT1G22690	Gibberellin-regulated fam.	5 13	2
AT1G23130	Lipid transport superfam.	5 13	2
AT1G28330	DRM1	5 13	2
AT1G28370	ERF11	12 13	2
AT1G30840	PUP4	5 13	2
AT1G31340	RUB1	2 3	2
AT1G49430	LRD2	5 13	2
AT1G50280	NPH3 fam.	12 13	2
AT1G51660	MKK4	5 13	2
AT1G51680	4CL1	12 13	2
AT1G53310	PPC1	5 13	2
AT1G53540	HSP20-like	8 11	2
AT1G54000	GDSL-like lipase	1 2	2
AT1G55020	LOX1	5 13	2
AT1G56150	SAUR-like	5 12	2
AT1G60140	TPS10	5 13	2
AT1G61340	F-box family	12 13	2
AT1G62300	WRKY6	2 12	2
AT1G62360	STM	2 9	2
AT1G64160	Dirigent-like	8 13	2
AT1G65390	PP2-A5	12 13	2
AT1G66800	Alcohol dehydrogenase	6 8	2
AT1G68640	PAN	1 2	2
AT1G70290	TPS8	5 12	2
AT1G73870	B-box type zinc finger	11 12	2
AT1G75440	UBC16	5 7	2
AT1G78580	TPS1	3 8	2
AT1G80840	WRKY40	2 12	2
AT2G01180	LPP1	12 13	2
AT2G02990	RNS1	3 4	2
AT2G03760	SOT12	7 11	2
AT2G17850	Cell cycle control phosphatase superfam.	5 8	2

AGI	Examples of regulated genes	References	No. of hits
AT2G18150	Peroxidase	1 2	2
AT2G18700	TPS11	5 7	2
AT2G19110	HMA4	7 13	2
AT2G19500	CKX2	2 8	2
AT2G19670	PRMT1A	4 6	2
AT2G22770	NAI1	6 8	2
AT2G22860	PSK2	3 5	2
AT2G22870	EMB2001	6 13	2
AT2G24540	AFR	11 13	2
AT2G25900	ATTZF1	5 13	2
AT2G28160	FIT1	5 8	2
AT2G28630	KCS12	7 13	2
AT2G30210	LAC3	12 13	2
AT2G30520	RPT2	11 13	2
AT2G33840	Tyrosyl-tRNA synthetase	12 13	2
AT2G34600	JAZ7	7 13	2
AT2G36870	XTH32	6 13	2
AT2G38620	CDKB1;2	5 12	2
AT2G38940	PHT1;4	5 13	2
AT2G40000	HSPRO2	12 13	2
AT2G40610	EXP8	1 2	2
AT2G40970	MYBC1	3 8	2
AT2G41010	CAMBP25	12 13	2
AT2G41100	TCH3	1 2	2
AT2G43140	bHLH superfam.	8 10	2
AT2G46400	WRKY46	8 12	2
AT2G46790	APRR9	5 11	2
AT2G47260	WRKY23	3 8	2
AT2G47890	B-box type zinc finger	5 13	2
AT2G47990	SWA1	4 6	2
AT2G48140	EDA4	12 13	2
AT3G01090	KIN10	3 12	2
AT3G01550	PPT2	5 11	2
AT3G02760	Class II aaRS superfam.	7 13	2
AT3G05200	ATL6	12 13	2
AT3G08770	LTP6	5 13	2
AT3G09600	LCL5	8 11	2
AT3G12270	PRMT3	4 6	2
AT3G12580	HSP70	4 6	2
AT3G15030	TCP4 / MEE35	4 6	2
AT3G15210	ERF4	12 13	2
AT3G15570	NPH3 fam.	6 12	2
AT3G16720	ATL2	12 13	2
AT3G16770	ERF72	2 5	2
AT3G18680	Amino acid kinase fam.	12 13	2
AT3G18780	ACT2	2 9	2
AT3G22370	AOX1A	5 13	2
AT3G23630	IPT7	2 10	2
AT3G24290	AMT1;5	3 8	2
AT3G44260	CAF1A	12 13	2
AT3G48520	CYP94B3	11 13	2
AT3G48750	CDKA;1	2 9	2
AT3G49240	EMB1796	4 6	2
AT3G49530	NTL6	12 13	2
AT3G49660	WDR5A	6 12	2
AT3G50060	MYB77	12 13	2
AT3G50970	LTI30	5 13	2
AT3G54220	SCR	2 5	2
AT3G55500	EXP16	6 13	2
AT3G55510	RBL	4 6	2
AT3G55980	SZF1	12 13	2
AT3G56400	WRKY70	2 8	2
AT3G56990	EDA7	4 12	2
AT3G58660	L1p/L10e fam.	4 6	2
AT3G59400	GUN4	11 13	2
AT3G60390	HAT3	5 13	2
AT3G60690	SAUR-like	12 13	2
AT3G61190	BAP1	12 13	2
AT3G61630	CRF6	12 13	2
AT3G61850	DAG1	1 2	2

AGI	Examples of regulated genes	References	No. of hits
AT3G63440	CKX6	2 12	2
AT4G00050	UNE10	11 13	2
AT4G03510	RMA1	5 13	2
AT4G04610	APR1	7 13	2
AT4G04940	WD-40 repeat fam.	4 6	2
AT4G10120	ATSPS4F	3 11	2
AT4G12400	HOP3	7 11	2
AT4G13750	NOV	4 6	2
AT4G14540	NF-YB3	1 2	2
AT4G15680	Thioredoxin	6 8	2
AT4G15690	Thioredoxin	7 10	2
AT4G17460	HAT1	5 13	2
AT4G17500	ERF-1	12 13	2
AT4G18780	CESA8	3 5	2
AT4G19680	IRT2	8 13	2
AT4G21120	AAT1	8 13	2
AT4G23800	HMG box	12 13	2
AT4G23810	WRKY53	2 3	2
AT4G25410	bHLH superfam.	8 10	2
AT4G28250	EXPB3	8 13	2
AT4G30270	SEN4	11 13	2
AT4G30800	OB-fold-like	4 6	2
AT4G31320	SAUR-like	8 13	2
AT4G31800	WRKY18	12 13	2
AT4G33070	Pyruvate decarboxylase	5 13	2
AT4G33420	Peroxidase	10 13	2
AT4G35480	RHA4B	12 13	2
AT4G35770	SEN1	5 13	2
AT4G36040	DnaJ-domain superfam.	7 13	2
AT4G36360	BGAL3	6 13	2
AT4G36430	Peroxidase	1 2	2
AT4G37260	MYB73	7 13	2
AT4G37790	HAT22	5 12	2
AT4G38470	STY46	5 12	2
AT4G39770	TPPH	8 10	2
AT5G02490	HSP70-2	6 12	2
AT5G05690	DWF3	5 13	2
AT5G06800	myb-like	12 13	2
AT5G07460	PMSR2	12 13	2
AT5G07580	ERF B3 subfam.	10 13	2
AT5G12110	EF1B	6 8	2
AT5G14070	ROXY2	10 13	2
AT5G15850	COL1	5 11	2
AT5G15970	KIN2	1 2	2
AT5G19260	FAF3	6 8	2
AT5G19730	Pectin lyase-like	6 7	2
AT5G19890	Peroxidase	3 8	2
AT5G22410	RHS18	8 13	2
AT5G23000	MYB37	1 2	2
AT5G25810	TINY	5 12	2
AT5G38020	SAMT-like	6 8	2
AT5G40390	RS5	8 13	2
AT5G44120	CRA1	11 12	2
AT5G44210	ERF9	5 13	2
AT5G45340	CYP707A3	12 13	2
AT5G47230	ERF5	12 13	2
AT5G47240	NUDT8	7 13	2
AT5G49450	BZIP1	7 13	2
AT5G49730	FRO6	5 11	2
AT5G50915	bHLH superfam.	6 8	2
AT5G51190	ERF B3 subfam.	12 13	2
AT5G52640	HSP83	4 6	2
AT5G55700	BMY6	12 13	2
AT5G56030	ERD8	4 6	2
AT5G56270	WRKY2	2 7	2
AT5G56860	GNC	6 12	2
AT5G57090	PIN2	3 5	2
AT5G57180	CIA2	4 6	2
AT5G57620	MYB36	1 2	2
AT5G59820	ZAT12	12 13	2

AGI	Examples of regulated genes	References	No. of hits
AT5G61600	ERF104	12 13	2
AT5G62430	CDF1	11 13	2
AT5G63780	SHA1	7 13	2
AT5G64920	CIP8	5 13	2
AT5G65730	XTH6	3 11	2
AT1G01120	KCS1	5	1
AT1G03020	Thioredoxin	8	1
AT1G03360	RRP4	6	1
AT1G03630	PORC	5	1
AT1G04220	KCS2	5	1
AT1G04310	ERS2	5	1
AT1G04940	TIC20	4	1
AT1G05560	UGT1	11	1
AT1G06080	ADS1	5	1
AT1G06230	GTE4	12	1
AT1G07640	OBP2	5	1
AT1G08090	ATNRT2.1	5	1
AT1G08810	MYB60	5	1
AT1G09250	bHLH superfam.	5	1
AT1G09570	PHYA	5	1
AT1G10480	ZFP5	8	1
AT1G11260	STP1	5	1
AT1G12420	ACR8	12	1
AT1G13260	RAV1	5	1
AT1G13570	F-box/RNI-like superfam.	12	1
AT1G14550	Peroxidase	8	1
AT1G14920	GAI	5	1
AT1G15100	RHA2A	12	1
AT1G15250	Zinc-binding ribosomal protein fam.	6	1
AT1G15580	IAA5	1	1
AT1G16060	ADAP	2	1
AT1G18140	LAC1	8	1
AT1G18500	IPMS1	12	1
AT1G18570	HIG1	12	1
AT1G19440	KCS4	5	1
AT1G20930	CDKB2;2	5	1
AT1G23740	AOR	11	1
AT1G23870	TPS9	5	1
AT1G25550	myb-like fam.	7	1
AT1G26560	BGLU40	6	1
AT1G26770	EXP10	8	1
AT1G27050	ATHB54	6	1
AT1G27730	ZAT10	12	1
AT1G29690	CAD1	12	1
AT1G29930	LHCB1.3	9	1
AT1G30080	Glycosyl hydrolase superfam.	8	1
AT1G30135	JAZ8	11	1
AT1G30370	alpha/beta-Hydrolases superfam.	12	1
AT1G31163	F-box associated ubiquitination effector fam.	7	1
AT1G31860	HISN2	6	1
AT1G35580	CINV1	5	1
AT1G36370	SHM7	7	1
AT1G44350	ILL6	5	1
AT1G44760	Adenine nucleotide alpha hydrolases-like	12	1
AT1G47230	CYCA3;4	12	1
AT1G49620	ICK5	6	1
AT1G49780	PUB26	12	1
AT1G50500	HIT1	12	1
AT1G51700	DOF1	12	1
AT1G52890	NAC019	11	1
AT1G54050	HSP20-like	11	1
AT1G56500	Haloacid dehalogenase-like hydrolase fam.	12	1
AT1G58290	HEMA1	11	1
AT1G59218	CC-NBS-LRR class	7	1

AGI	Examples of regulated genes	References	No. of hits
AT1G59500	GH3.4	5	1
AT1G61580	RPL3B	4	1
AT1G62975	bHLH superfam.	7	1
AT1G64230	UBC28	5	1
AT1G64660	MGL	5	1
AT1G64780	AMT1;2	5	1
AT1G64860	SIG1	11	1
AT1G66100	PR-13 fam.	11	1
AT1G66330	Ssenescence-associated fam.	11	1
AT1G67030	ZFP6	8	1
AT1G67100	LBD40	5	1
AT1G67810	SUFE2	12	1
AT1G68520	B-box type zinc finger	7	1
AT1G68670	myb-like fam.	7	1
AT1G68840	RAP2.8	12	1
AT1G68880	BZIP8	8	1
AT1G69920	GSTU12	7	1
AT1G70000	myb-like	12	1
AT1G71692	XAL1	5	1
AT1G71880	SUC1	5	1
AT1G72520	LOX4	12	1
AT1G72930	TIR	5	1
AT1G72940	TIR domain fam.	12	1
AT1G73500	MKK9	7	1
AT1G73830	BEE3	7	1
AT1G74560	NRP1	6	1
AT1G77380	AAP3	8	1
AT1G77570	Winged helix-turn-helix repressor	4	1
AT1G77760	NR1	5	1
AT1G78090	TPPB	8	1
AT1G78240	TSD2	12	1
AT2G01570	RGA1	5	1
AT2G02710	PLP	7	1
AT2G02950	PKS1	5	1
AT2G05100	LHCB2.1	9	1
AT2G14610	PR-1	11	1
AT2G16365	F-box fam.	12	1
AT2G18300	bHLH superfam.	5	1
AT2G18470	PERK4	1	1
AT2G18660	PNP-A	11	1
AT2G19060	SGNH hydrolase-type esterase superfam.	8	1
AT2G20100	bHLH superfam.	8	1
AT2G20142	TIR domain fam.	12	1
AT2G21045	Cell cycle control phosphatase superfam.	5	1
AT2G21060	GRP2B	5	1
AT2G21950	SKIP6	5	1
AT2G23050	MEL4	8	1
AT2G23170	GH3.3	10	1
AT2G23290	MYB70	7	1
AT2G23680	WCOR413 fam	12	1
AT2G23810	TET8	12	1
AT2G26010	PDF1.3	8	1
AT2G26040	PYL2	5	1
AT2G26980	CIPK3	5	1
AT2G28950	EXPA6	6	1
AT2G29540	RPC14	6	1
AT2G29560	ENOC	12	1
AT2G30130	ASL5	8	1
AT2G30810	Gibberellin-regulated fam.	3	1
AT2G32220	L27e protein fam	4	1
AT2G35930	PUB23	12	1
AT2G36640	ECP63	5	1
AT2G37430	Zinc finger superfam.	5	1
AT2G38120	AUX1	5	1
AT2G38390	Peroxidase	1	1
AT2G38470	WRKY33	12	1
AT2G38540	LTP1	1	1

AGI	Examples of regulated genes	References	No. of hits
AT2G39700	EXPA4	8	1
AT2G40100	LHCB4.3	5	1
AT2G40140	CZF1	12	1
AT2G40940	ERS1	12	1
AT2G40940	EDA40	12	1
AT2G40950	bZIP17	12	1
AT2G41230	ORS1	8	1
AT2G42530	COR15B	11	1
AT2G42540	COR15A	11	1
AT2G43650	EMB2777	6	1
AT2G44070	Translation initiation factor	3	1
AT2G44680	CKB4	12	1
AT2G45000	EMB2766	12	1
AT2G47270	UPB1	5	1
AT2G47520	ERF71	5	1
AT2G47550	Pectin methylesterase inhibitor superfam.	12	1
AT2G47590	PHR2	5	1
AT2G47800	EST3	8	1
AT3G02240	RGF7	8	1
AT3G02550	LBD41	5	1
AT3G02885	GASA5	8	1
AT3G04060	NAC046	5	1
AT3G04420	NAC048	8	1
AT3G04530	PPCK2	10	1
AT3G04740	SWP	5	1
AT3G05040	HST1	7	1
AT3G05690	UNE8	7	1
AT3G05770	F10A16.6	8	1
AT3G06850	BCE2	5	1
AT3G07390	AIR12	5	1
AT3G07750	3'-5'-exoribonuclease fam	6	1
AT3G07770	HSP89.1	6	1
AT3G08660	NPH3 fam.	7	1
AT3G09220	LAC7	12	1
AT3G09270	GSTU8	8	1
AT3G09440	HSP70 fam.	4	1
AT3G09520	EXO70H4	12	1
AT3G10490	NAC052	12	1
AT3G10580	Homeodomain-like superfam.	7	1
AT3G10710	RHS12	5	1
AT3G12110	ACT11	5	1
AT3G12560	TRFL9	7	1
AT3G12670	EMB2742	6	1
AT3G13450	DIN4	5	1
AT3G14050	RSH2	12	1
AT3G15540	IAA19	1	1
AT3G15720	Pectin lyase-like	12	1
AT3G15950	NAI2	8	1
AT3G16530	Lectin like	5	1
AT3G17170	RFC3	6	1
AT3G17465	RPL3P	6	1
AT3G18000	XPL1	12	1
AT3G18030	HAL3	12	1
AT3G19850	NPH3 fam.	5	1
AT3G20770	EIN3	5	1
AT3G21110	PUR7	6	1
AT3G21150	EIP6	12	1
AT3G21890	B-box type zinc finger	11	1
AT3G22425	IGPD	12	1
AT3G23150	ETR2	5	1
AT3G23830	GRP4	6	1
AT3G25780	AOC3	5	1
AT3G25790	myb-like fam.	8	1
AT3G25900	HMT-1	12	1
AT3G25940	TFIIB zinc-binding	6	1
AT3G27170	CLC-B	11	1
AT3G27950	GDSL-like Lipase superfam.	5	1
AT3G29030	EXP5	6	1

AGI	Examples of regulated genes	References	No. of hits
AT3G30220	ATL4	1	1
AT3G43190	SUS4	5	1
AT3G44550	FAR5	8	1
AT3G44890	RPL9	12	1
AT3G44990	XTR8	8	1
AT3G45060	NRT2.6	5	1
AT3G45140	LOX2	5	1
AT3G45260	C2H2-like zinc finger	12	1
AT3G45300	IVD	5	1
AT3G46130	MYB48	5	1
AT3G46230	HSP17.4	11	1
AT3G46740	MAR1	12	1
AT3G47340	ASN1	5	1
AT3G48360	BT2	7	1
AT3G49220	Pectin methylesterase inhibitor superfam.	12	1
AT3G49670	BAM2	6	1
AT3G50260	CEJ1	12	1
AT3G50700	IDD2	7	1
AT3G51895	SULTR3;1	11	1
AT3G52340	SPP2	12	1
AT3G52430	PAD4	12	1
AT3G53310	AP2-like subfam. B3	7	1
AT3G54720	AMP1	6	1
AT3G55970	JRG21	9	1
AT3G57070	Glutaredoxin	12	1
AT3G57260	PR-2	11	1
AT3G57530	CPK32	5	1
AT3G58070	GIS	6	1
AT3G58190	LBD29	10	1
AT3G59060	PIF5	12	1
AT3G59900	ARGOS	5	1
AT3G60140	DIN2	5	1
AT3G61880	CYP78A9	10	1
AT3G62550	Adenine nucleotide alpha hydrolases-like	10	1
AT3G62950	Thioredoxin	9	1
AT3G63310	BIL4	12	1
AT3G63460	WD-40 repeat fam.	12	1
AT4G02200	Drought-responsive fam.	12	1
AT4G05410	YAO	4	1
AT4G05460	F-box/RNI-like superfam.	5	1
AT4G08040	ACS11	11	1
AT4G08780	Peroxidase	8	1
AT4G08950	EXO	8	1
AT4G09760	Choline synthase	5	1
AT4G12550	AIR1A	1	1
AT4G12570	UPL5	12	1
AT4G13390	EXT12	5	1
AT4G13510	AMT1;1	5	1
AT4G14560	IAA1	1	1
AT4G14940	AO1	5	1
AT4G15290	CSLB05	5	1
AT4G15880	ESD4	12	1
AT4G16070	Lipase, class 3	7	1
AT4G16350	CBL6	5	1
AT4G16780	HAT4	5	1
AT4G17280	Auxin-responsive fam.	8	1
AT4G17490	ERF6	12	1
AT4G17900	PLATZ fam.	12	1
AT4G18880	HSF A4A	12	1
AT4G21760	BGLU47	8	1
AT4G21830	MSRB7	4	1
AT4G22080	RHS14	5	1
AT4G22200	AKT2	8	1
AT4G22590	TPPG	5	1
AT4G23190	CRK11	5	1
AT4G24040	TRE1	5	1
AT4G25480	CBF3	8	1
AT4G25700	BCH1	12	1

AGI	Examples of regulated genes	References	No. of hits
AT4G25810	XTR6	11	1
AT4G25910	NFU3	12	1
AT4G26200	ACS7	12	1
AT4G26430	CSN6B	7	1
AT4G27030	FAD4	11	1
AT4G27590	Heavy metal transport superfam.	6	1
AT4G28450	DWD motif	6	1
AT4G28850	XTH26	5	1
AT4G29130	HXK1	5	1
AT4G31120	SKB1	6	1
AT4G32980	ATH1	5	1
AT4G33220	PME44	6	1
AT4G33790	FAR3	5	1
AT4G34160	CYCD3	9	1
AT4G34520	KCS18	7	1
AT4G34750	SAUR-like	5	1
AT4G34770	SAUR-like	5	1
AT4G35090	CAT2	11	1
AT4G36650	PBRP	12	1
AT4G36740	ATHB40	1	1
AT4G36740	ATHB40	1	1
AT4G36990	HSF4	5	1
AT4G37220	Cold acclimation (WCOR413) fam.	5	1
AT4G37390	AUR3	7	1
AT4G37610	BT5	7	1
AT4G37770	OPR2	5	1
AT4G37770	ACS8	5	1
AT4G37980	CAD7	5	1
AT4G38850	SAUR-AC1	3	1
AT4G38860	SAUR-like	5	1
AT4G39640	GGT1	3	1
AT5G02180	Amino acid transporter fam.	11	1
AT5G03310	SAUR-like	8	1
AT5G03610	GDSL-like Lipase superfam.	7	1
AT5G03730	CTR1	12	1
AT5G03740	HD2C	12	1
AT5G04140	GLS1	12	1
AT5G04770	CAT6	11	1
AT5G05850	PIRL1	12	1
AT5G06000	EIF3G2	6	1
AT5G06300	Lysine decarboxylase fam.	8	1
AT5G07440	GDH2	5	1
AT5G08600	U3 ribonucleoprotein fam.	8	1
AT5G08620	STRS2	6	1
AT5G10230	ANNAT7	8	1
AT5G10250	DOT3	11	1
AT5G10720	CKI2	8	1
AT5G10970	Zinc finger superfam.	8	1
AT5G12020	HSP17.6II	11	1
AT5G13220	JAS1	11	1
AT5G13330	RAP2.6L	5	1
AT5G13740	ZIF1	2	1
AT5G13910	LEP	8	1
AT5G14520	Pescadillo-related	6	1
AT5G15210	ATHB30	6	1
AT5G15830	BZIP3	8	1
AT5G16240	Stearoyl-acyl-carrier-protein desaturase fam.	5	1
AT5G16690	ORC3	7	1
AT5G16750	TOZ	6	1
AT5G17790	VAR3	12	1
AT5G18170	GDH1	5	1
AT5G18670	BMY3	11	1
AT5G18820	EMB3007	8	1
AT5G20230	SAG14	5	1
AT5G20280	SPS1F	7	1

AGI	Examples of regulated genes	References	No. of hits
AT5G20320	DCL4	12	1
AT5G20830	SUS1	5	1
AT5G20930	TSL	3	1
AT5G23190	CYP86B1	8	1
AT5G24120	SIG2	11	1
AT5G24210	alpha/beta-hydrolases superfam.	12	1
AT5G24520	TTG1	5	1
AT5G24580	Heavy metal transport superfam.	6	1
AT5G24660	LSU2	7	1
AT5G25350	EBF2	5	1
AT5G26030	FC1	12	1
AT5G26920	CBP60G	12	1
AT5G37020	ARF8	5	1
AT5G37500	GORK	7	1
AT5G38410	Rubisco small subunit 3b	1	1
AT5G38940	RmlC-like cupins superfam	8	1
AT5G39850	Ribosomal protein S4	4	1
AT5G40480	EMB3012	6	1
AT5G41315	GL3	7	1
AT5G41410	BEL1	12	1
AT5G41800	Amino acid transporter fam.	12	1
AT5G43370	PHT1;2	5	1
AT5G45080	PP2-A6	8	1
AT5G46330	FLS2	5	1
AT5G46710	PLATZ fam.	12	1
AT5G46760	MYC3	12	1
AT5G48070	XTH20	8	1
AT5G48150	PAT1	5	1
AT5G49020	PRMT4A	12	1
AT5G49270	DER9	12	1
AT5G49520	WRKY48	12	1
AT5G50740	Heavy metal transport superfam.	6	1
AT5G50760	SAUR-like	8	1
AT5G51070	SAG15	5	1
AT5G51310	MWD22.26	5	1
AT5G51460	TPPA	11	1
AT5G51780	bHLH superfam.	12	1
AT5G52100	CRR1	12	1
AT5G52310	COR78	11	1
AT5G53890	PSKR2	12	1
AT5G53950	CUC2	1	1
AT5G54080	HGO	5	1
AT5G54370	LEA protein-related	5	1
AT5G55050	GDSL-like lipase superfam.	8	1
AT5G55690	MADS-box fam.	7	1
AT5G56550	OXS3	7	1
AT5G57220	CYP81F2	12	1
AT5G57900	SKIP1	5	1
AT5G57980	RPB5C	7	1
AT5G58430	EXO70B1	12	1
AT5G60020	LAC17	12	1
AT5G60780	NRT2.3	5	1
AT5G61900	BON1	12	1
AT5G62440	DOMINO1	6	1
AT5G63450	MLE2.8	11	1
AT5G64730	WD40 repeat-like superfam.	7	1
AT5G65980	Auxin efflux carrier fam.	3	1
AT5G67030	ABA1	11	1
AT5G67480	BT4	7	1

1. Che P, Gingerich DJ, Lall S, Howell SH: Global and hormone-induced gene expression changes during shoot development in *Arabidopsis*. *Plant Cell* 2002, 14:2771-2785.
2. Hoth S, Ikeda Y, Morgante M, Wang X, Zuo J, Hanafey MK, Gaasterland T, Tingey SV, Chua NH: Monitoring genome-wide changes in gene expression in response to endogenous cytokinin reveals targets in *Arabidopsis thaliana*. *FEBS Lett* 2003, 554:373-380.
3. Rashotte AM, Carson SD, To JP, Kieber JJ: Expression profiling of cytokinin action in Arabidopsis. *Plant Physiol* 2003, 132:1998-2011.
4. Kiba T, Aoki K, Sakakibara H, Mizuno T: *Arabidopsis* response regulator, *ARR22*, ectopic expression of which results in phenotypes similar to the *wol* cytokinin-receptor mutant. *Plant Cell Physiol* 2004, 45:1063-1077.
5. Brenner WG, Romanov GA, Köllmer I, Bürkle L, Schmülling T: Immediate-early and delayed cytokinin response genes of *Arabidopsis thaliana* identified by genome-wide expression profiling reveal novel cytokinin-sensitive processes and suggest cytokinin action through transcriptional cascades. *Plant J* 2005, 44:314-333.
6. Kiba T, Naitou T, Koizumi N, Yamashino T, Sakakibara H, Mizuno T: Combinatorial microarray analysis revealing *Arabidopsis* genes implicated in cytokinin responses through the His->Asp phosphorelay circuitry. *Plant Cell Physiol* 2005, 46:339-355.
7. Rashotte AM, Mason MG, Hutchison CE, Ferreira FJ, Schaller GE, Kieber JJ: A subset of *Arabidopsis* AP2 transcription factors mediates cytokinin responses in concert with a two-component pathway. *Proc Natl Acad Sci USA* 2006, 103:11081-11085.
8. Lee DJ, Park JY, Ku SJ, Ha YM, Kim S, Kim MD, Oh MH, Kim J: Genome-wide expression profiling of *ARABIDOPSIS* RESPONSE REGULATOR (ARR7) overexpression in cytokinin response. *Mol Genet Genomics* 2007, 277:115-137.
9. Taniguchi M, Sasaki N, Tsuge T, Aoyama T, Oka A: ARR1 directly activates cytokinin response genes that encode proteins with diverse regulatory functions. *Plant Cell Physiol* 2007, 48:263-277.
10. Yokoyama A, Yamashino T, Amano Y, Tajima Y, Imamura A, Sakakibara H, Mizuno T: Type-B ARR Transcription Factors, ARR10 and ARR12, are Implicated in Cytokinin-Mediated Regulation of Protoxylem Differentiation in Roots of *Arabidopsis thaliana*. *Plant Cell Physiol* 2007, 48:84-96.
11. Argyros RD, Mathews DE, Chiang Y-H, Palmer CM, Thibault DM, Etheridge N, Argyros DA, Mason MG, Kieber JJ, Schaller GE: Type B response regulators of Arabidopsis play key roles in cytokinin signaling and plant development. *Plant Cell* 2008, 20:2102-2116.
12. Heyl A, Ramireddy E, Brenner WG, Riefler M, Allemeersch J, Schmülling T: The transcriptional repressor ARR1-SRDX suppresses pleiotropic cytokinin activities in Arabidopsis. *Plant Physiol* 2008, 147:1380-1395.
13. Brenner WG, Schmülling T: Transcript profiling of cytokinin action in *Arabidopsis* roots and shoots discovers organ-specific cytokinin responses. *BMC Plant Biology* 2012, 12:112.
